# Centhaquine Restores Renal Blood Flow and Protects Tissue Damage After Hemorrhagic Shock and Renal Ischemia

**DOI:** 10.3389/fphar.2021.616253

**Published:** 2021-05-03

**Authors:** Amaresh K. Ranjan, Zhong Zhang, Seema Briyal, Anil Gulati

**Affiliations:** ^1^ Chicago College of Pharmacy, Midwestern University, Downers Grove, IL, United States; ^2^ Pharmazz Inc. Research and Development, Willlowbrook, IL, United States

**Keywords:** acute kidney injury, hypoxia inducible factors, shock, resuscitation, hemorrhage, centhaquine

## Abstract

**Background:** Centhaquine (CQ) (Lyfaquin^®^) is in late stage clinical development as a safe and effective first-in-class resuscitative agent for hemorrhagic shock patients (NCT02408731, NCT04056065, and NCT04045327). Acute kidney injury (AKI) is known to be associated with hemorrhagic shock. Hence, effect of CQ on protection of kidneys from damage due to hemorrhagic shock was investigated.

**Methods:** To assess effect of CQ on AKI in shock, we created a rat model with hemorrhagic shock and AKI. Renal arteries were clamped and de-clamped to induce AKI like ischemia/reperfusion model and hemorrhage was carried out by withdrawing blood for 30 min. Rats were resuscitated with CQ (0.02 mg/kg) for 10 min. MAP, heart rate (HR), and renal blood flow (RBF) were monitored for 120 min.

**Results:** CQ produced a significant improvement in RBF compared to vehicle (*p*< 0.003) even though MAP and HR was similar in CQ and vehicle groups. Blood lactate level was lower (*p* = 0.0064) in CQ than vehicle at 120 min post-resuscitation. Histopathological analysis of tissues indicated greater renal damage in vehicle than CQ. Western blots showed higher HIF-1α (*p* = 0.0152) and lower NGAL (*p* = 0.01626) levels in CQ vs vehicle. Immunofluorescence in the kidney cortex and medulla showed significantly higher (*p*< 0.045) expression of HIF-1α and lower expression of Bax (*p*< 0.044) in CQ. Expression of PHD 3 (*p*< 0.0001) was higher, while the expression of Cytochrome C (*p* = 0.01429) was lower in the cortex of CQ than vehicle.

**Conclusion:** Results show CQ (Lyfaquin^®^) increased renal blood flow, augmented hypoxia response, decreased tissue damage and apoptosis following hemorrhagic shock induced AKI, and may be explored to prevent/treat AKI.

**Translational Statement:** Centhaquine (CQ) is safe for human use and currently in late stage clinical development as a first-in-class resuscitative agent to treat hemorrhagic shock. In the current study, we have explored a novel role of CQ in protection from hemorrhagic shock induced AKI, indicating its potential to treat/prevent AKI.

## Introduction

Blood loss in excess may lead to hemorrhagic/hypovolemic shock, due to insufficient blood circulation, insufficient tissue perfusion and poor oxygenation (Gutierrez et al., 2004). Hemorrhagic/hypovolemic shock remains a major cause of mortality among severe trauma patients (Tisherman et al., 2015). Mortality in these patients in initial stage is known to be linked to excessive blood loss and later to multiple organ failures (Bonanno, 2011). At present, recommended non-surgical treatments for hypovolemic shock include rapid volume resuscitation to restore the intravascular volume and ventilation to increase oxygen supply. However, an aggressive resuscitation to increase intravascular volume and oxygenation cause a rapid increase in blood pressure and hyperoxemia which could be more damaging to organs. A controlled fluid resuscitation with small volume has been advocated (Carrick et al., 2016); however, mortality is still high and many patients have associated multiple organ failure. Among organs that fail, acute kidney failure (AKI) is most frequent (Legrand et al., 2010; Harrois et al., 2018). Kidneys are important regulatory organs for maintaining the plasma homeostasis by filtration and reabsorption. Moreover, it is also at the center stage of blood pressure regulation. After hemorrhage, kidney microvascular oxygen level starts to drop at a much earlier stage than other organs such as the gut or heart (Legrand et al., 2010) and acute injury follows. A damaged and compromised condition of kidneys in shock leads to further disturbance in the homeostasis, which accelerates failures of other organs and may ultimately cause death (Faubel, 2009; Okusa, 2010; Grams and Rabb, 2012). Therefore, a resuscitative agent that could protect kidneys from damage following hemorrhagic shock would significantly improve clinical outcome of patients with hemorrhagic shock.

**FIGURE 1:**
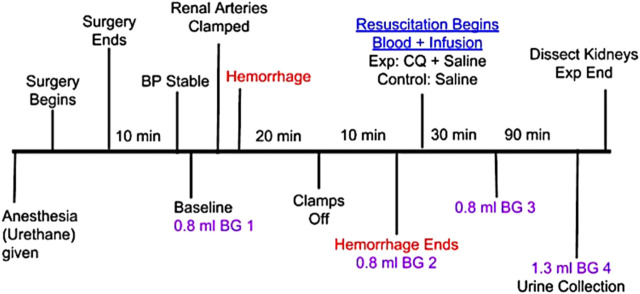
Schematic representation of *in vivo* experiment in rats involving hypovolemic shock, acute kidney injury with kidney ischemia/reperfusion (I/R) and resuscitation with CQ. Procedure from anesthesia to the end of the *in vivo* experiment is shown with brief description of each step and time intervals of some important steps. B.G.–indicates a small volume blood collection for analyzing blood gas components.

Centhaquin (INN: Centhaquine) (World Health Organisation, 2019) (2-[2-(4-(3-methyphenyl)-1-piperazinyl)]ethyl-quinoline) citrate is in late stage clinical development (NCT02408731, NCT04056065, and NCT04045327) as a new resuscitative agent for hemorrhagic shock. Pre-clinical studies of centhaquine (CQ) have demonstrated its superiority over commonly used resuscitative agents in reducing mortality following hypovolemic shock (Gulati et al., 2012; Lavhale et al., 2013; Papapanagiotou et al., 2016). The clinical studies have shown its safety, tolerability and efficacy in treating hemorrhagic shock (Gulati, 2016; Gulati et al., 2020; Gulati et al., 2020) and recently it received marketing authorization from Indian regulatory agency. We carried out the current study to explore the role of CQ on kidney perfusion and protection of kidney tissues against hypoxic damage. We created a rat model with “hemorrhagic shock and kidney ischemia/reperfusion (KI/R)” by inducing AKI in the hemorrhagic shock rat model used in our previous studies (Gulati et al., 2012; Lavhale et al., 2013). The rats were resuscitated with CQ (0.02 mg/kg) or vehicle to assess the effect of CQ (Figure 1, a diagrammatic representation of the procedure).

## Results

**FIGURE 2:**
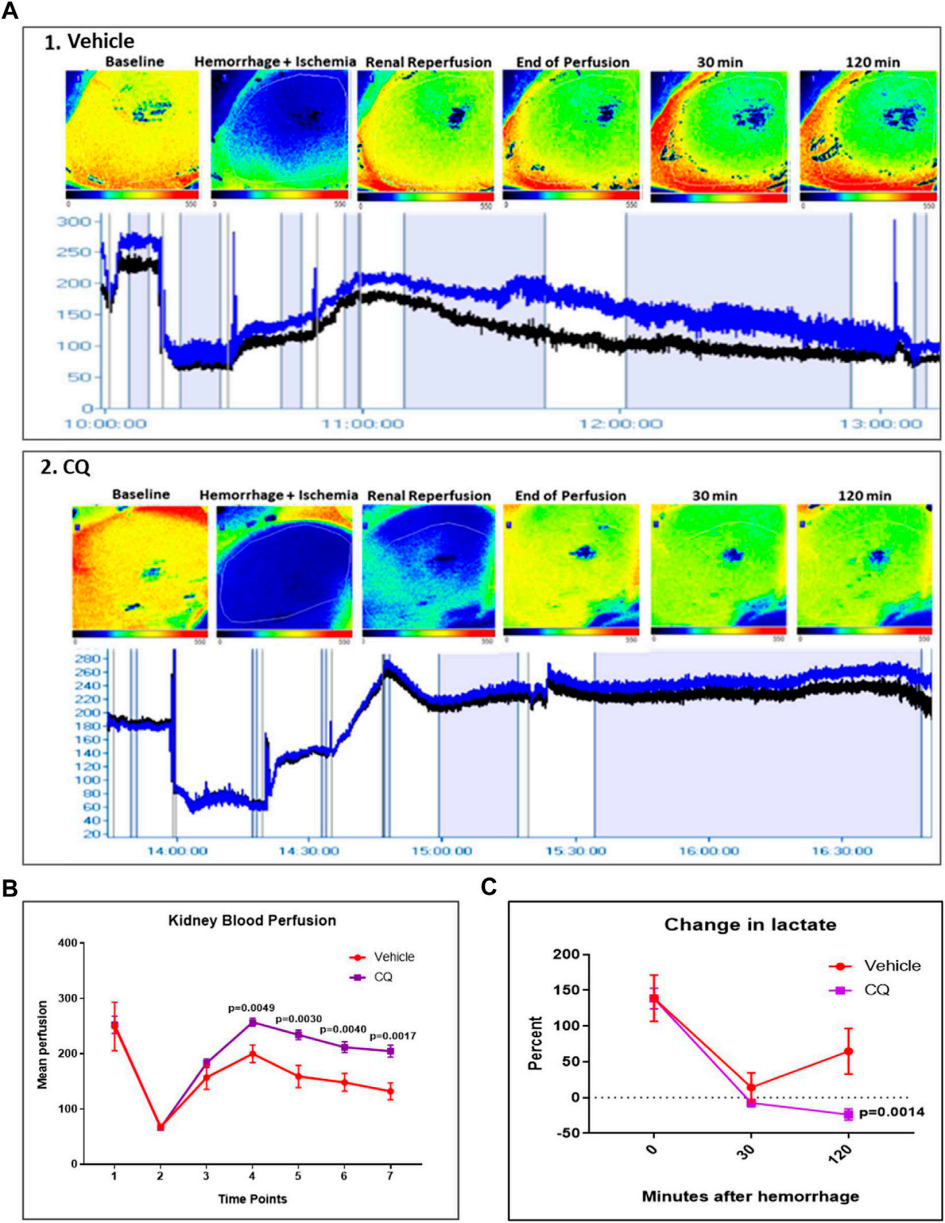
Effect of CQ on kidney blood flow in a rat model of kidney ischemia/reperfusion with hypovolemic shock. **(A)**, 1 and 2, representative images of blood flow in kidneys of vehicle 1) and CQ 2). Upper panel in 1 and 2 shows kidney images obtained from PeriCam PSI blood flow imaging system. Red represents highest blood flow and blue represents the lowest. The lower panels in 1 and 2 show the blood flow graphs–Blue line indicates blood flow in complete imaged area, Black line indicates blood flow in the kidney. *N* = 7 for vehicle and *N* = 8 for CQ. **(B)**, a graphical representation of kidney blood flow at different time points (1–baseline, 2–after hemorrhage, 3–after de-clamping renal arteries, 4–0 min post-perfusion, 5–30 min post-perfusion, 6–90 min post-perfusion and 7–120 min post-perfusion). Data represents mean ± SEM values. **(C)**, the graph represents percent change in blood lactate levels in CQ and vehicle rats at different time points–after hemorrhage (0 min of resuscitation), at 30 and 120 min of resuscitation. Baseline is indicated with dotted lines. Data represents mean ± SEM values. *N* = 7 for vehicle and *N* = 8 for CQ. Statistical analysis–unpaired *t*-tests.

Effect of resuscitation with CQ on blood perfusion in kidneys and blood lactate. Blood flow in kidneys decreased from mean perfusion value (MPV) of 250 to 50 after hemorrhage and KI [Figures 2A,B, time point 2 (hemorrhage) vs. 1 (baseline)], while de-clamping of renal arteries (reperfusion) showed improvement (MPV 50 to 150) in kidney blood flow in both groups of rats (Figures 2A,B, time point 3 vs 2). After hemorrhage and KI/R, resuscitation with CQ or vehicle was carried out and kidney blood flow was measured. Resuscitation with CQ showed significant improvement in kidney blood flow compared to that of NS at 10 min (Figure 2B, time point 4, *p* = 0.0049), 30 min (Figure 2B, time point 5, *p* = 0.003), 90 min (Figure 2B, time point 6, *p* = 0.004) and 120 min (Figure 2B, time point 7, *p* = 0.0017) (Supplementary Table S1) post-resuscitation. These results indicate that resuscitation with CQ significantly improved kidney blood perfusion compared to saline resuscitation.

Blood lactate level was equally elevated in both vehicle and CQ rats after hemorrhage, indicating similar extent of hemorrhagic shock in both group of rats. Following resuscitation, lactate levels were significantly lower in rats treated with CQ than vehicle (Figure 2C).

**FIGURE 3:**
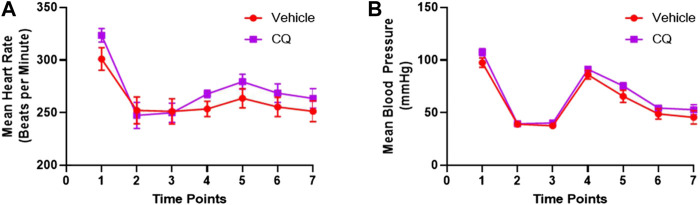
Effect of CQ on cardiovascular activities, HR and MAP. **(A, B)**, a graphical representation of mean HR **(A)** and MAP **(B)** at different time points (1–baseline, 2–after hemorrhage, 3–after de-clamping renal arteries, 4–0 min post-perfusion, 5–30 min post perfusion, 6–90 min post-perfusion and 7–120 min post-perfusion). Data represents mean ± SEM values. *N* = 7 for vehicle and *N* = 8 for CQ. Statistical analysis–unpaired *t*-tests.

Effect of CQ on cardiovascular actions in the rat model of hemorrhage and KI/R. A significant decrease in heart rate and mean arterial blood pressure (MAP) was observed in both CQ and NS groups of rats after kidney ischemia and hemorrhage (Figures 3A,B, time point 2 compared to 1). No change in these parameters was observed after de-clamping of renal arteries while hemorrhage was continued (Figures 3A,B, time point 3 compared to 2). After 10 min of resuscitation, no significant change in heart rate (HR) was observed (Figure 3A, time point 4 compared to 3) (Supplementary Table S2) while a significant improvement in MAP (Figure 3B, time point 4 compared to 3) (Supplementary Table S3) was observed in both groups of rats. The HR and MAP were observed at similar level in both CQ and NS rats at every time points of the experiment (Figures 3A,B). These results suggest that CQ and NS resuscitation had similar effects on HR and MAP after KI/R and hemorrhage.

**FIGURE 4:**
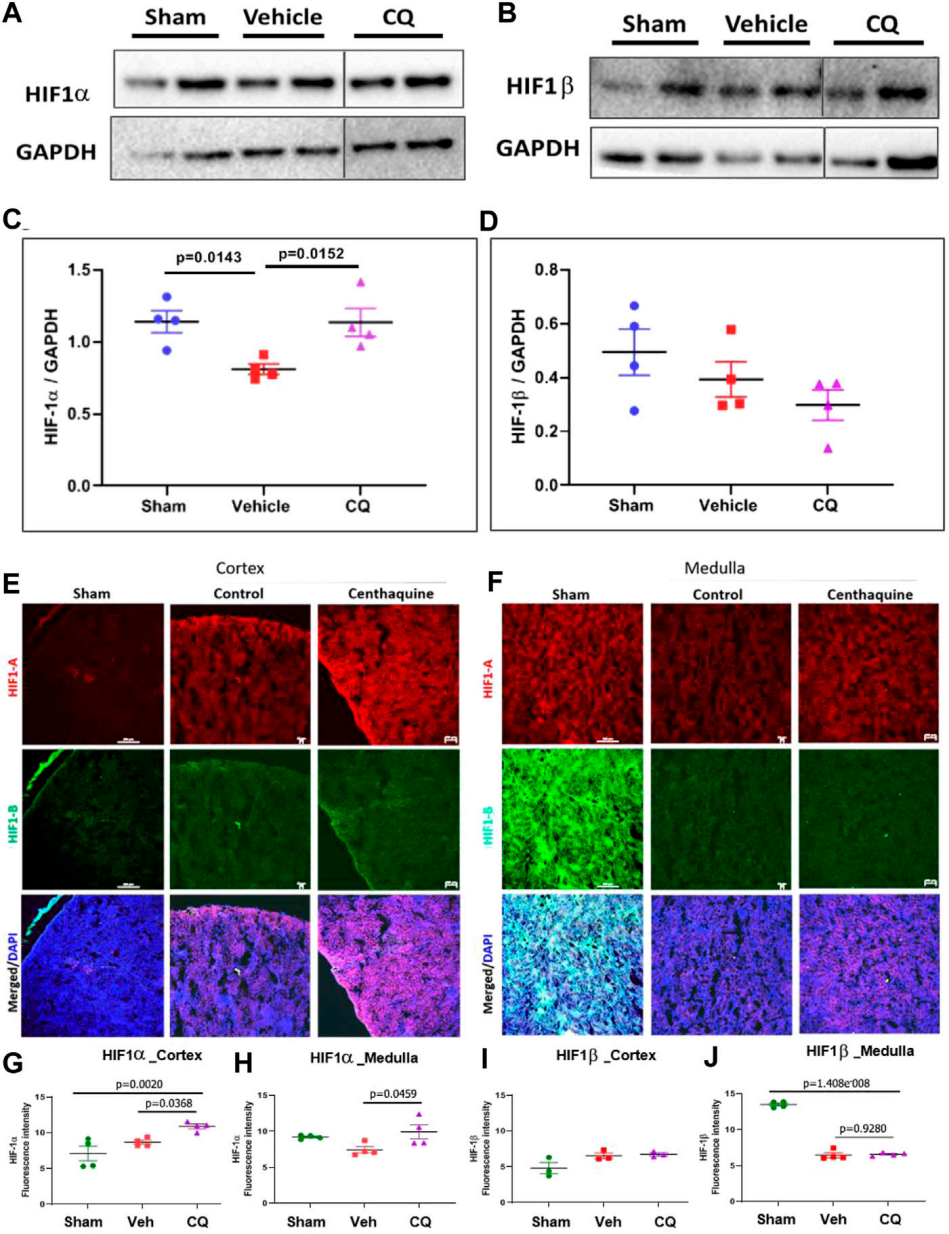
Effect of CQ on expression of hypoxia responsive factors in kidneys. A and B, representative western blots of HIF-1α **(A)** and HIF-1β **(B)** and their respective GAPDH (loading control). **(C, D)**, graphical representation of HIF-1α/GAPDH **(C)** and HIF-1β/GAPDH (**D**) normalized data. The error bar represents mean ± SEM. *N* = 4. Black vertical lines in A and B show that the bands were cropped from the same blot (full blots are provided as Supplementary Figures S1, S2). Statistical analysis Ordinary One Way ANOVA and Fisher’s test. E and F, representative images of immunofluorescence of HIF-1α (red) and HIF-1β (green) in cortex **(E)** and medulla **(F)** in sham, vehicle (control) and CQ. **(G–J)**, graphs of mean fluorescence intensity of HIF-1α in cortex (**G**) and medulla **(H)**, and of HIF-1β in cortex **(I)** and medulla **(J)**. *N* = 4. Nuclei in all immunofluorescence microscopy images were stained with DAPI (blue). Statistical analysis- Ordinary One Way ANOVA and Fisher’s test.

**FIGURE 5:**
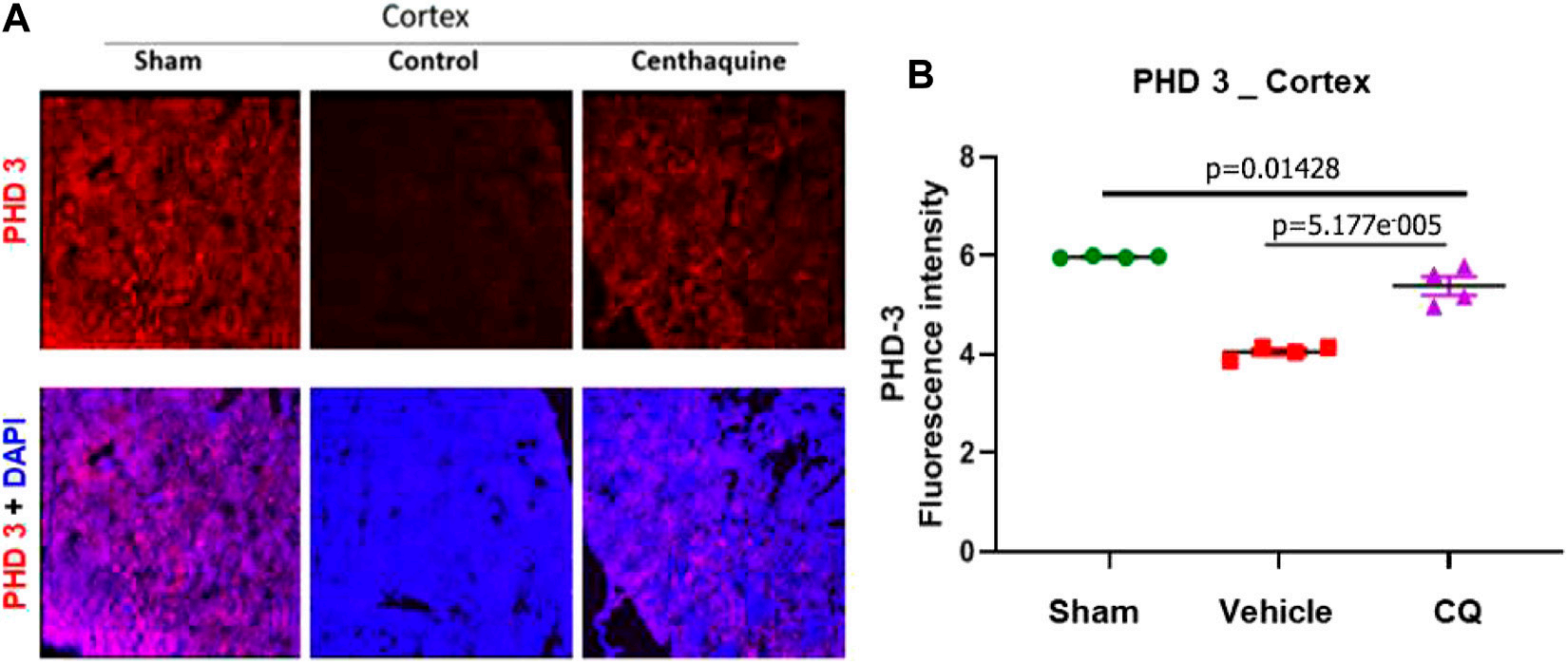
Expression of hypoxia responsive factor, PHD 3 in kidney cortex. **(A)**, representative images of immunofluorescence of PHD 3 (red) in cortex **(A)** in sham, vehicle (control) and CQ. **(B)**, a graph of mean fluorescence intensity of PHD 3 in cortex. *N* = 4. Nuclei in all immunofluorescence microscopy images were stained with DAPI (blue). Statistical analysis Ordinary One Way ANOVA and Fisher’s test.

Effect of CQ on the expression of hypoxia responsive survival factors. Western blot analysis was carried out to assess the expression of hypoxia responsive proteins HIF-1α and HIF-1β in kidney tissues (Figures 4A,B; Supplementary Figures S1, S2) Significantly higher expression of HIF-1α was seen in CQ rat kidney tissues compared to vehicle (Figures 4A,C). We also observed significantly decreased level of HIF-1α in vehicle rat kidney tissues compared to that of sham rats, while no significant difference in HIF-1α in CQ was observed compared to sham (Figure 4C). On the other hand, the expression of HIF-1β was similar in kidney tissues of both groups (Figures 4B,D). Kidney tissues of sham, vehicle and CQ were also analyzed with immunofluorescence to further delineate the expression of the hypoxia related factors in cortical and medullary regions of the kidney. Expression of HIF-1α was significantly higher in CQ compared to vehicle in both cortical and medullary regions of kidneys (Figures 4E–H). The HIF-1α expression was also higher in CQ than sham in the cortical but not in the medullary region of kidneys (Figures 4E–H). While on the other hand expression HIF-1β was similar in CQ compared to NS rats in both cortical and medullary regions (Figures 4E–J). We also examined the expression of oxygen dependent HIF regulatory protein, PHD 3. It was significantly upregulated in the cortex of CQ compared to vehicle rats; however, it was lower than sham (Figures 5A,B). PHD 3 was not detectable in medullary kidney tissues of vehicle, CQ or sham rats (data not shown). These results indicate that CQ could initiate hypoxia responsive survival signaling in kidney tissues after hemorrhage and KI/R.

**FIGURE 6:**
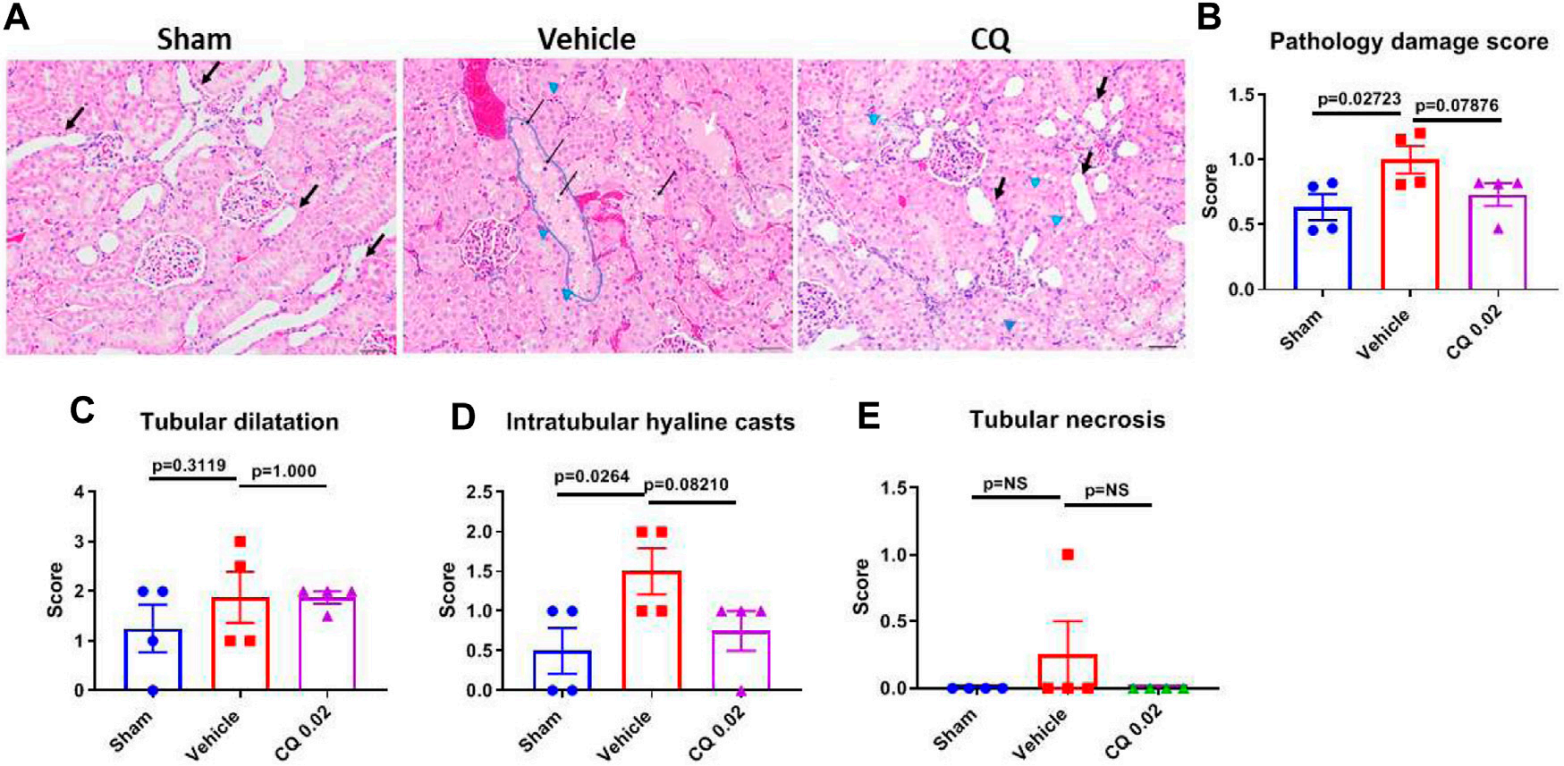
Effect of CQ on kidney tissue damage. **(A)**, representative microscopy images of H and E stained kidney tissue sections from sham, saline and CQ treated rats. Some of the dilated cortical tubules are marked with “black arrows”, vacuoles (clear spaces) in tubules are shown by “blue arrow heads”, a highly vacuolated tubule is “encircled with blue line”, necrotic cells are indicated by “thin black arrows” and tubules with hyaline casts are shown with “white arrows”. Bar scale = 50 µm. **(B)**, a graph showing pathological damage score of the kidney tissues in sham, vehicle and CQ treated rats, Calculated on the basis of scores of tubular dilatation **(C)**, hyaline casts **(D)** and tubular necrosis or degeneration **(E)**. Error bars represent mean ± SEM. *N* = 4. Statistical analysis- Ordinary One Way ANOVA and Fisher’s test.

**FIGURE 7:**
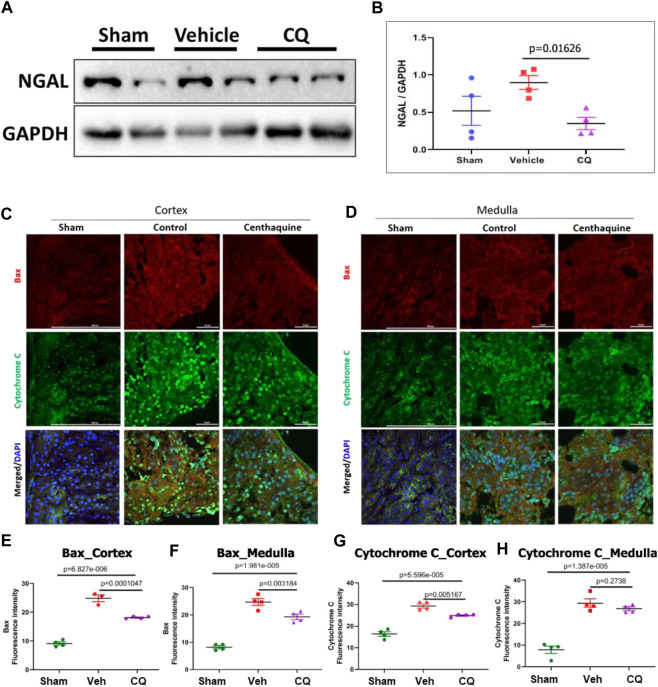
Effect of CQ on expression of kidney damage markers. **(A)**, representative western blots of acute kidney damage marker, NGAL and GAPDH (loading control). Black vertical lines in A show that the bands were cropped from the same blot (full blots are provided as Supplementary Figure S3). **(B)**, graphical representation of NGAL/GAPDH normalized data. The error bar represents mean ± SEM. *N* = 4. **(C, D)**, representative images of immunofluorescence of apoptotic proteins, Bax (red) and Cytochrome C (green) in cortex **(C)** and medulla **(D)** in sham, vehicle (control) and CQ. **(E–H)**, graphs of mean fluorescence intensity of Bax in cortex **(E)** and medulla **(F)**, and of Cytochrome C in cortex **(G)** and medulla **(H)**. *N* = 4. Nuclei in all immunofluorescence microscopy images were stained with DAPI (blue). Statistical analysis- Ordinary One Way ANOVA and Fisher’s test.

Effect of CQ on renal damage and apoptosis in kidneys. Damage in rat kidney tissues was assessed by hematoxylin and eosin (H&E) staining and histopathological analysis. Pathology Damage Score (PDS) in vehicle was significantly higher (*p* = 0.0272) than sham kidneys, while in CQ it was lower than vehicle; however, the difference was not significant compared to either vehicle (*p* = 0.0787) or sham (Figures 6A,B). PDs was calculated based on scores of tubular dilatation, hyaline casts and necrosis (Figures 6C–E). Kidney tissues were also analyzed for expression of kidney damage markers including the early kidney damage marker, NGAL using western blots. The expression of NGAL was significantly reduced in CQ compared to vehicle rats, (Figures 7A,B; Supplementary Figure S3). Expression of other kidney damage markers NAG 2, HFABP, Cystatin C, and TIM 1 and apoptotic marker Cytochrome c was similar in the lysate of whole kidney tissues of rats of all the groups (Supplementary Figure S4). Expression of apoptotic and kidney damage markers was also tested in the cortical and medullary regions of kidneys using immunofluorescence. Significantly decreased expression of apoptotic marker, Bax in cortex as well as in medulla of kidneys of CQ rats compared to vehicle was seen (Figures 7C–F); however, its expression in both cortex and medulla was significantly higher than that of sham (Figures 7C–F, red). Expression of another apoptotic marker, Cytochrome c was significantly lower only in the cortex of CQ rat kidneys than that of vehicle (Figures 7C,D,G,H). Expression of kidney damage markers HFABP, TIM 1, Cystatin C and NAG 2 showed no significant change in the cortex and medulla of CQ and vehicle rat kidneys (Supplementary Figures S5, S6). These results suggest that resuscitation with CQ helps in protecting kidneys against acute damage by decreasing apoptosis.

### Effect of CQ on Mitochondrial DNA

**FIGURE 8:**
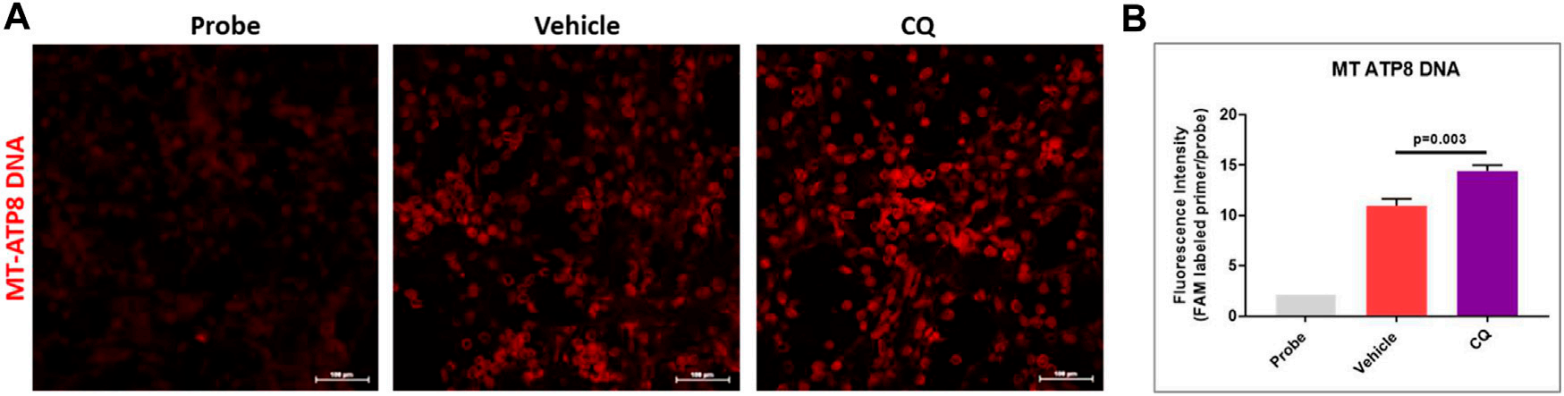
Effect of CQ on mitochondrial DNA in kidneys. **(A)**, representative *in situ* PCR images vehicle and CQ treated rat kidney tissues after 120 min of resuscitation. Probe represents the negative control (background fluorescence) of the *in situ* PCR reaction. Red fluorescence indicates amplified MT-ATP8 DNA in mitochondria. Image magnification 200x. **(B)**, fluorescence intensity graph of MT-ATP8 DNA. *N* = 4. Values are expressed as mean ± SEM. Statistical analysis- Ordinary One Way ANOVA and Fisher’s test.

Mitochondrial DNA analysis using *in situ* PCR in kidney tissues of CQ and vehicle treated rats was carried out to assess the potential ROS mediated mitochondrial DNA damage after hemorrhage/resuscitation. We observed significantly higher mitochondrial DNA amount (fluorescence) in CQ treated rats than that of vehicle rats (Figure 8A,B), which could be because of reduced effect of ROS mediated mitochondrial DNA damage in CQ than vehicle.

## Discussion

Loss of excessive blood causes hypovolemic/hemorrhagic shock, which could be aggravated with loss of homeostasis and electrolyte imbalance (hyperkalemia), acid-base imbalance (base deficit), decrease in pH (acidosis), increase in lactate (lactate or metabolic acidosis), change in pO2 and pCO2 levels, etc (Haljamäe, 1985; Haljamae, 1993). It has been reported that kidney is among the frontline organ which gets affected most severely after hemorrhagic/hypovolemic shock (Legrand et al., 2010; Harrois et al., 2018). Since, kidney is an important regulatory organ for maintaining plasma (blood) homeostasis including pH balance by filtration and reabsorption, and blood pressure (Wadei and Textor, 2012), protection of kidneys after shock would help in restoring homeostasis and preventing further complications such as multiple organ failure.

Our group is developing CQ as a new resuscitative agent to be used as a frontline adjuvant to standard of care for patients with hypovolemic shock. Our studies have demonstrated its effectiveness compared to control in reducing mortality following hypovolemic shock in animal models as well as in patients (Gulati et al., 2012; Gulati et al., 2013; Lavhale et al., 2013; Papapanagiotou et al., 2016; Gulati et al., 2020). Interestingly, we also observed the effect of CQ on reduction in serum creatinine more significantly than that of control. Creatinine level decreased from 0.78 ± 0.08 mg/dl at baseline to 0.54 ± 0.04 (*p* = 0.0277) at end of study in control and from 1.16 ± 0.12 at baseline to 0.68 ± 0.05 (*p* = 0.0021) at end of the study in CQ cohort (Gulati et al., 2019), which indicates the potential role of CQ in protection of kidneys from damage during hemorrhagic shock.

In the current study, we used a rat model of hemorrhagic shock and induced AKI with clamping renal arteries to induce ischemic damage to kidneys along with hemorrhagic shock. Clamping of renal arteries and hemorrhage was started simultaneously. After 20 min, renal arteries were de-clamped to make a model similar to kidney ischemia/reperfusion (KI/R) of acute injury (Ko et al., 2017; Wang et al., 2017). To increase the severity of hemorrhagic shock, hemorrhage was continued for 10 more minutes after de-clamping renal arteries. Rats were resuscitated with either CQ or normal saline (vehicle) and their effects were compared. CQ showed improvement in kidney blood flow better than vehicle and resuscitation with CQ was more effective in reducing blood lactate level than vehicle. In comparison to vehicle, CQ showed no significant difference in MAP and HR in this study, while our previous preclinical and clinical studies have shown significant improvement in MAP after CQ resuscitation than control (Gulati et al., 2012; Gulati et al., 2013; Lavhale et al., 2013; Papapanagiotou et al., 2016). Since, kidneys were exposed to severe ischemic condition prior to resuscitation in this study, kidney damage could be attributing to these results and further studies are needed to confirm the role of kidneys in CQ mediated cardiovascular actions. Nonetheless, a study by Zakaria et al. has shown that even though the intravascular resuscitation with crystalloid could improve cardiovascular activities they were not enough to improve the blood flow in splanchnic organs after hemorrhage (Zakaria et al., 2003). Similarly, we observed significantly improved renal blood flow after resuscitation with CQ than vehicle, although HR and MAP were similar in both groups. These observations indicate that an improvement in kidney blood flow by CQ may not be associated with an increase in blood pressure but it may have an effect on the microcirculatory system.

The microcirculatory system consists of complex network of small diameter (<100–150 µm) blood vessels e.g. arterioles, venules and capillaries and plays an important role in regulating oxygen supply to meet the metabolic needs of tissues. In homeostasis, they are systemically regulated by sympathetic and parasympathetic nervous system (Safar and Struijker-Boudier, 2010; Sheng and Zhu, 2018) However, in pathological or shock conditions, circulation in these vessels is compromised and a substantial volume of blood is pooled in the microcirculatory system (Bonanno, 2011). These microcirculatory system is enriched in α adrenergic receptors. It has been shown that CQ acts on venous α2B adrenergic receptors to produce constriction and increase venous blood return to the heart and also stimulates central α2A adrenergic receptors to produce a decrease in systemic vascular resistance (SVR) (Gulati et al., 2020; Gulati et al., 2020) and thus it helps in increasing the cardiac output. Kidney tissues have abundant α-adrenergic receptors and their localization is primarily in renal arterioles, glomeruli and tubules of the renal cortex and medulla (Jackson and Insel, 1993). They are involved in the regulation of renal blood flow, glomerular filtration rate, renin release and sodium re-absorption (de Leeuw and Birkenhager, 1988). Presumably, CQ mediated α-adrenergic signaling in kidneys would be helping in restoring the blood flow after AKI and hemorrhage. Also, it appears that an increase in renal blood flow is independent of an increase in blood pressure and cardiac output, hence we are suggesting that CQ induced increase in renal blood flow could be due to a selective renal vasodilation.

Our previous studies demonstrated contrasting effects of CQ at high and low doses, and it is used as a hypotensive agent at high dose (0.2–0.45 mg/kg), while as a resuscitative agent at low dose (0.01–0.05 mg/kg) (Srimal et al., 1990; Gulati et al., 1991; Gulati et al., 1993; Gulati et al., 2012; Gulati et al., 2013; Lavhale et al., 2013; Reniguntala et al., 2015; Kontouli et al., 2019; Gulati et al., 2020). Based on the hemorrhagic shock study, we tested a low dose of 0.02 mg/kg and a high dose of 0.2 mg/kg in our current study to examine which dose is effective in protecting kidneys from acute injury. The results indicated no significant changes in 0.2 mg/kg CQ dose compared to vehicle in various important parameters (e.g., kidney blood flow, HIFs and apoptosis) tested in this study (Supplementary Figure S7).

Change in tissue blood flow after hemorrhage is known to alter oxygen supply in organs, which leads to change in cellular metabolism with partial catabolism of glucose to produce lactate instead of CO2 and H2O. Excessive production of lactate causes lactic acid (metabolic) acidosis, which is an important parameter for determining patients’ condition and known to be correlated with death in shock patients (Kimmoun et al., 2015; Filho et al., 2016). We observed several fold increase in blood lactate level in rats of all the experimental groups after hemorrhage, which comes down near to baseline at 30 min of post resuscitation, however resuscitation with vehicle didn’t provide sustainable effect and at 120 min post resuscitation the lactate level in NS was observed significantly higher than CQ. Lactate is also known to induce hypoxia inducible factors (HIFs) (Lee et al., 2015; Laustsen et al., 2019). HIF1 is a dimeric protein and plays important role in oxygen homeostasis in tissues. HIF1 consists of HIF-1α and HIF-1β subunits (Ziello et al., 2007). HIF-1α is shown to be oxygen labile and has been inversely related to oxygen level in tissues, while HIF-1β has been reported as a constitutive protein (Iyer et al., 1998; Semenza, 2001). HIF-1α is known to be most active during short periods (2–24 h) of intense hypoxia and drives the initial response to hypoxia; however, during chronic hypoxic exposure, it is HIF-2α that plays the major role in driving the hypoxic response (Holmquist-Mengelbier et al., 2006; Koh et al., 2011). The HIF-1α to HIF-2α switch is known to be important for their divergent but complementary roles during the hypoxic response of tissues under physiological and pathophysiological conditions. Since our study mimicked the acute kidney injury setting and involved a short period (2 h after resuscitation) of recovery after hemorrhage and kidney injury, HIF-1α was a suitable candidate to study the effect of centhaquine resuscitation in these animals. Moreover, the importance of HIF-1α in hypoxia has also been demonstrated in regulating the expression of PHD-2 as well as PHD-3, which generates an auto-regulatory loop controlling HIF-1α stability under hypoxia and reoxygenation (Marxsen et al., 2004). In hypoxic condition, the hydroxylation activity of PHD-3 as well as PHD-2 is reduced, and HIF-1α is protected from degradation. Thus, abundance of HIF-1α in hypoxia is increased. HIF-1α acts as a transcription factor for the PHDs genes and facilitates their expression and starts the auto-regulatory loop, which leads to simultaneous increased abundance of HIF-1α and PHD-3 at a stage of hypoxic condition as it prepares cells for adapting the condition of re-oxygenation following ischemia. Conversely, the injury could cause more damage to tissues when the “auto-regulatory loop” is perturbed and the stability or abundance of HIF-1α is compromised in the hypoxic condition. In kidneys of vehicle treated rats, lower levels of HIF-1α and PHD-3 compared to that of sham rats were observed. While on the other hand CQ helped in maintaining the “auto-regulatory loop” in the injured kidney tissues and the levels of HIF-1α and PHD-3 were similar to that of sham.

The regulation of HIF1 A level is mediated through PHD 1–3 (Prolyl Hydroxylase-4 Domain 1–3). PHDs hydroxylase HIF1 1α in presence of oxygen and facilitate its ubiquitination and degradation (Wu et al., 2018). Although among PHD 1-3, PHD 2 is known to be most effective in regulating oxygen dependent HIF1 1α degradation, We chose PHD-3 in our study, because apart from hydroxylation and degradation of HIF-1α, it is also known to act as a cofactor for HIF-1α recruitment on hypoxia response elements (HREs) within Vegfa, Slc2a1, and Ldha promoters in hypoxia (Schoepflin et al., 2017), which suggests that PHD3 is critical for stabilizing binding of HIF-1α to the promoters of a select set of target genes and activates their transcription in hypoxic condition. Thus PHD-3 helps in regulating the expression of the angiogenic factor; VEGF, glucose transporter; GLUT-1, and glycolytic enzyme; lactate dehydrogenase. These factors are known to play important roles in tissue perfusion and cellular metabolism adaptation for ATP production in hypoxic condition, which could determine the cellular fate following ischemic injury/damage. Moreover, it is also known to hydroxylate and promote E3 ligase mediated ubiquitination and degradation of β2 adrenergic receptors (Xie et al., 2009), while CQ is known to act on alpha-adrenergic receptors. Since α and β adrenergic receptors play a pivotal role in regulating the blood flow, cardiac output, blood pressure and bronchodilation, the role of PHD-3 after CQ resuscitation in shock and ischemia would be important for drug development in future.

During shock and tissue injury reactive oxygen species (ROS) generation is also known to affect the stabilization of HIF-1α. Although we have not measured the ROS in our study, yet we assessed the mitochondrial DNA using *in situ* PCR technique in kidney tissues of the rats. Since ROS generation mostly takes place in mitochondria and known to cause mitochondrial DNA damage (Nissanka and Moraes, 2018). We assessed the mitochondrial DNA using mitochondrial DNA specific TaqMan probe and primers for MT-ATP8 gene, which is present in mitochondrial genome as a single copy and is absent in nuclear genome. We performed *in situ* PCR in kidney tissues and carried out fluorescence confocal microscopy to detect the FAM labeled TaqMan probe as described in our previous studies (Ranjan et al., 2012; AK Ranjan and Hardikar, 2015; Ranjan et al., 2020). We observed that CQ had significantly higher mitochondrial DNA fluorescence than vehicle, which could be due to less ROS generation in CQ than vehicle (Figure 8).

We further analyzed kidney tissue for expression of kidney damage markers and apoptotic markers. We observed, a significant decrease in early kidney damage marker, NGAL in CQ than that NS treated rats. While we observed no significant difference in the expression of another early AKI marker, cystatin C among all the groups tested in this study. The comparative study in post-operative AKI patients has shown that change in NGAL and cystatin C could be detected in patients’ serum as early as 2 h after operation, while change in creatinine was not detected in even at 24 h post operation (Hekmat & Mohebi, 2016). The study has also shown that NGAL was more sensitive (90.2 vs. 79.2%) and specific (89.5 vs. 78.5%) than cystatin C in early diagnosis (Zhang et al., 2017). Also, it has been shown that NGAL expression indicates apoptotic stress in epithelial cells (Han et al., 2018), hence decrease NGAL expression in CQ may indicate a decrease in apoptosis of kidney tubular epithelial cells. We also analyzed other kidney damage markers such as HFABP, NAG 2 and TIM 1; however, no significant difference in their level in CQ and vehicle was observed (Supplementary Figures S4–S6). This could be because the kidney injury in our study was for very short span of time (20 min of ischemia and hemorrhage and 10 min of only hemorrhage) and after resuscitation animals were observed for 120 min, which might not be sufficient to have effect of damage on expression of these markers in kidneys.

We also evaluated expression of apoptotic marker, Bax and cytochrome C in these samples. Both, Bax and cytochrome C are essential components in mitochondria dependent apoptosis pathway. We observed significantly reduced level of Bax in CQ cortical and medullary regions of kidneys than that of NS treated rats. Significantly reduced level of cytochrome C was seen in the cortical region, while in medullary region it decreased but could not reach the level of statistical significance. To examine the renal function, we assessed the levels of creatinine and BUN in the rat plasma at baseline and at 120 min after hemorrhage and resuscitation. We observed significantly higher levels of creatinine and BUN in vehicle and CQ than sham rats; however, similar levels of creatinine and BUN in vehicle and CQ were observed at 120 min (Supplementary Figure S8). The recovery period after resuscitation following ischemic damage was short (2 h), which could be the reason of no significant change in these markers in CQ rats compared to vehicle. Further studies with longer recovery period are required to evaluate the kidney function improvement in these rats.

Thus, the results of the study indicate that resuscitation with CQ helps in protection of kidney tissues by increasing tissue blood perfusion and hypoxia inducible factors mediated anti-apoptotic and survival responses. Based on the results of the study, the potential mechanisms of CQ could be enhancement of blood flow in kidneys after acute injury and anti-apoptotic role mediated via hypoxia responsive factors activation, which would be promoting angiogenesis and metabolic adaptation in injured tissues for their survival and function.

## Conclusion

Overall, our study has demonstrated a novel role of resuscitation with CQ in improving kidney blood flow, hypoxia survival response and protection against acute kidney injury. Further studies on other kidney damage models (e.g., septic shock, diabetes, antibiotics, etc.) will help to prove its potential to develop as a new drug for acute kidney injury.

## Methods

To explore the role of CQ on kidney perfusion and protection of kidney tissues against hypoxic damage, we performed CQ resuscitation in a rat model with hemorrhagic shock and kidney ischemia/reperfusion (KI/R). To induce AKI in hemorrhagic shock condition, renal arteries of rats were clamped, and hemorrhage was carried out. After shock and renal ischemia, resuscitation with CQ (0.02 mg/kg) or vehicle was carried out to assess the effect of CQ (Figure 1, a diagrammatic representation of the procedure). Kidney blood flow and arterial blood gases were determined. Histopathological evaluation, western blots, and immunofluorescence analyses of kidneys to assess tissue damage, apoptosis and hypoxia responsive factors were carried out.

### Animal Care and Experimentation

The animal experiment protocol was approved by the Institutional Animal Care and Use Committee of Midwestern University (IACUC protocol 2502). Male Sprague-Dawley rats with an average weight of 288 g were purchased from Envigo, Indianapolis, IN. All rats were acclimatized to for at least 4 days with proper care and readily available food and water. *Guidelines*: Results are reported according to the ARRIVE (Animal Research: Reporting of *In vivo* experiments) guideline listed in EQUATOR Network library. *Ethical aspects:* All animal care and use for experimental procedures were performed in accordance with the guidelines of the Institutional Animal Care and Use Committee (IACUC) of Midwestern University. All biohazards were handled in accordance with Midwestern University Bio-safety Committee standard operating procedures and in compliance with OLAW/OSHA regulations.

### Hemorrhage and Kidney Ischemia/Reperfusion

Animals were anesthetized using urethane. Urethane was administered in two doses of 1.5 g/kg body weight via intraperitoneal injection. Urethane was chosen as the anesthetic agent because it produces long lasting (8–10 h) anesthesia with minimal cardiovascular and respiratory system depression. After anesthesia, surgery was carried out to open the abdominal area and renal arteries were located. Right kidney was exposed to assess blood flow using a Laser Doppler probe (PERIMED PeriCam PSI camera, Inc., Ardmore, PA). A femoral artery was catheterized and the catheter was attached to the pressure monitoring probe of AD Instruments PowerLab (AD Instruments Inc., Colorado Springs, CO, United States). Continual mean arterial pressure and heart rate measurements were carried out throughout the experiment. The femoral artery in the other leg of the rat was catheterized to draw blood to induce hemorrhage and to collect blood samples for blood gas analyses. A femoral vein was catheterized and was used as an infusion site for resuscitative agents. Following the surgery and catheterization, heparin (1 U/g body wt) was infused through femoral vein and 10 min of stabilization period was provided. After stabilization of vital signs, 0.8 ml of blood was drawn from the femoral artery and 0.3 ml was used for blood gas analysis (first blood gas reading) and 0.5 ml was stored for future plasma assessments (the plasma isolated at this stage was used for baseline value). Both renal arteries were clamped using strings and hemorrhage was started, simultaneously. Hemorrhage was induced by manually withdrawing blood from the femoral artery at a rate of approximately 0.5–1 ml/min until a MAP between 35 and 40 mmHg was reached (MAP and HR were continuously monitored using a pressure transducer, AD instruments). A little volume of blood was infused back if MAP was fallen below 35 mmHg. A total of approximately 6.4 ml of blood from each rat was drawn, which is about 33% of the total blood volume of an adult rat weighing 280–300 gm. The MAP at 35–40 mmHg was maintained for the entire 30 min of hemorrhage by further withdrawal or infusion of blood if necessary. After completion of the hemorrhage, 0.8 ml of blood was withdrawn (0.3 ml for the second blood gas reading and 0.5 ml stored for the second plasma reading). For the sham treatment, rats were anesthetized similar to test rats and after anesthesia, surgery was carried out to open the abdominal area; however, no clamping of renal arteries was performed. Femoral arteries were catheterized to collect blood samples for blood gas analyses and monitoring mean arterial pressure as well as heart rate; however, no hemorrhage was carried out. The rats were monitored for the same time period of test rats.

### Resuscitation

The blood loss volume was calculated for each animal and the same volume was used for infusion with resuscitative agent. The infusion consisted of centhaquine (CQ) dissolved in saline (experimental groups) or saline (control group). An approximately 6.4 ml of CQ solution was infused in each of the test rats and same volume of saline was infused in vehicle or control rats. The rate of infusion was 1/10th volume of resuscitative agent per minute (total infusion time = 10 min). Thirty min post infusion (or 30 min after completion of resuscitation), 0.8 ml blood was drawn (0.3 ml for blood gas analysis and 0.5 ml stored for plasma as the third reading). At 120 min post infusion, 1.3 ml blood was drawn (0.3 ml for blood gas analysis and 1.0 ml stored for plasma as the fourth and final reading). At the end of experiment, urine was collected from the bladder and stored in the 4°C. The rats underwent bilateral nephrectomy. The right kidney (imaged) was stored in 10% formalin. And the left kidney was snap frozen in liquid nitrogen and stored in −80°C.

### Kidney Blood Flow Measurement

The kidney blood flow in the right kidney of each rat was measured using a PeriCam PSI system (PERIMED inc., Ardmore, PA). The system measures blood perfusion/flow through laser speckle contrast analysis (LSCA) technology in real time. Real time blood flow graph and video recording of the kidney flow both were recorded simultaneously and were analyzed using a software package “PIMSoft”. The whole kidney was selected as the ROI and blood flow at different time points (baseline −1, 0 min after hemorrhage −2, after de-clamping renal arteries −3, 0 min post-resuscitation −4, 30 min post resuscitation −5, 90 min post resuscitation −6 and 120 min post resuscitation −7) were analyzed by selecting an area on the graph at respective time points.

### Blood Gas Analysis

A portion of blood (0.3 ml) isolated at different time points of the experiments (baseline −1, 0 min after hemorrhage −2, 30 min after resuscitation −3, and 120 min post-resuscitation −4) were used for blood gas analysis. All blood gas samples were analyzed using a GEM Premier 3,000 blood gas analyzer (Instrumentation Laboratory, Bedford, MA). A number of blood components were analyzed including lactate, pH, pO2, pCO2, TCO2, HCO3, Hct, Hbc, Na^+^, K^+^, and Ca^++^ (Supplementary Tables S4, S5).

### Western Blots

Snap-frozen kidney tissues were homogenized in RIPA buffer (20 mM Tris-HCl pH 7.5, 120 mM NaCl, 1.0% Triton X100, 0.1% SDS, 1% sodium deoxycholate, 10% glycerol, 1 mM EDTA and 1X protease inhibitor, Roche). Homogenate was centrifuged at 12,000 RPM at 4°C for 30 min and supernatant was collected in a new tube. Folin-Ciocalteu’s Reagent was used to determine sample protein concentration. About 60 μg of protein from each sample was mixed with Laemmli sample buffer (Bio-Rad, Hercules, CA) and was denatured in a boiling water bath for 2 min. Proteins were resolved using a 10% SDS PAGE. The resolved proteins were transferred to a nitrocellulose membrane (Sigma-Aldrich, St. Louis, MO, United States), using a wet transfer method. 4% fat-free milk solution prepared in 1X TBST was used to block the membrane after completion of the transfer. Blocking was done at RT for 1 h. The membrane was incubated overnight with one of the primary antibodies anti-HIF1 A, HIF1 B, NGAL, HFABP, NAG2, TIM1, and Cystatin C at a dilution of 1:1,000 at 4°C overnight. The primary antibodies were purchased from Abcam, Cambridge, MA, United States. The membrane was washed three times with incubation of 10 min each at RT. The blot was incubated with respective secondary antibodies goat anti-rabbit or goat anti mouse IgG conjugated with horseradish peroxidase (HRP) at dilution of 1:10,000 for 2 h at room temperature. The secondary antibodies were purchased from Santa Cruz Biotech., Santa Cruz, CA, United States. Anti-GAPDH (Sigma-Aldrich, St. Louis, MO, United States) at dilution 1:10,000 was used to detect GAPDH as a loading control. The HRP chemiluminescence was developed with SuperSignal WestPico Chemiluminescent Substrate (Thermo Fisher Scientific, Bartlett, IL) and signals were acquired using the Bio-Rad gel documentation system XR (Bio-Rad, Hercules, CA). The protein band intensity was analyzed using ImageJ (NIH) software and graphs were plotted after normalizing with GAPDH expression.

### Immunofluorescence

Immunofluorescence technique was used to detect expression of hypoxia factors (HIF1 1α, HIF1 1β, and PHD3), kidney injury markers and apoptotic markers (Bax and Cytochrome C) in cortical and medullary regions of kidneys. A portion of snap-frozen kidneys was dissected out and submerged in 4% paraformaldehyde (PFA) prepared in 1X PBS and thawed at RT for 30 min. Kidney tissues were sliced into 10 µm using a cryostat (Microtome cryostat HM 505E; Walldorf, Germany) at −20°C. Further steps of immunofluorescence were followed with slight modifications as described in our previous studies (Briyal et al., 2015; Briyal et al., 2019). In brief, tissue sections were washed three times with 1X PBS and permeabilized with 1% Triton-x100 in PBS for 15 min at room temperature (RT). Blocking with 5% BSA in 1X PBS for 1 h at room temperature was carried out. The kidney sections were incubated with primary antibodies (1:200 diluted in 1X PBS) at 4°C overnight. Sections were washed twice in 1X PBS and incubated with Alexa Fluor 488 and/or 546-conjugated secondary antibodies (1:200, Abcam, Cambridge, MA) for 1 h at room temperature in the dark and mounted with prolong gold anti-fade reagent with DAPI (Cell Signaling Technology, Danvers, MA, United States). Fluorescence was detected using an inverted fluorescent microscope (Nikon Eclipse TiE, Melville, NY). All images for analysis were taken with the same exposure with a multi-channel ND acquisition using NIS Elements BR imaging software (Nikon Instruments, Inc., Melville, NY). Analyses was performed using NIS-Elements 3.01 imaging software from Nikon Instruments, Inc. (Melville, NY).

### Histopathology

The right kidneys of rats were used for histopathological analysis. At the end of the experiment (at 120 min after resuscitation) rats were sacrificed and the kidney was dissected out and preserved in 10% neutral buffered formalin solution and stored at room temperature. Formalin fixed kidneys were shipped to IDEXX BioAnalytics, Columbia, MO, for their histopathological analysis. The kidney samples were processed and analyzed by professional technicians at IDEXX BioAnalytics in a blinded manner. Briefly, the kidney tissue was embedded in paraffin wax and sectioned longitudinally with a microtome. Hematoxylin and eosin staining of the sections were carried out after deparaffinization. Coverslips were mounted and tissue sections were examined under the microscope at magnification of 20X. Tissue sections from different regions of each kidney were randomly selected and examined for various parameters including tubular dilatation, intratubular hyaline casts and tubular necrosis. These parameters were used to calculate the pathology damage score of kidneys.

### *In situ* PCR

*In situ* PCR was carried out to assess the mitochondrial DNA in the rat kidney tissues, with following our previously developed *in situ* PCR protocol (Ranjan et al., 2012; AK Ranjan and Hardikar, 2015). In brief, the fixed kidney tissues were cryo-sectioned (10 µm thick). Tissue sections were placed on 1% gelatin coated glass slides. Tissue section were permeabilized using freeze-thaw technique. Tissue sections were covered with sterile molecular grade (MG) water (150–200 µL) drops and incubated in −80°C for 5 min for freezing. The frozen section was quickly thawed at hot plate at 40 °C and the freeze thaw cycle was repeated 3–4 times. Blocking solution was prepared by adding 1% BSA in sterile MG water. Sections were incubated in the blocking solution at RT for 20 min. Tissue sections were washed once with sterile molecular grade water and 25 µL of TaqMan Master mix was added to each tissue section [For 300 µL of TaqMan Master mix–75 µL of 2X cDNA reaction mix (Cat # QP9001, Alkali Scientific, Fort Lauderdale, FL) 15 µL of TaqMan MT-ATP8 probe/primer (Cat # 4448489, ThermoFisher Scientific, Grand Island, NY) and 210 µL of 20% glycerol in molecular grade water were mixed.] A coverslip was carefully placed, and the slide was sealed with a scotch tape from all sides to prevent evaporation of the reagent. The slides were placed over an aluminum foil with coverslip facing down. A moist tissue paper on top of the slides were placed and the aluminum foil was wrapped around. The wrapped slides were placed in a thermal cycler with coverslip facing down and following PCR cycle was set up. PCR cycle–Step 1.95°C for 5.5 min, cycle 1. Step 2.95°C for 30 s, 57°C for 20 s, 72°C for 40 s, cycle–39. Step 3.72°C for 2 min, cycle −1. Step 4.7°C for ∞. After PCR cycles, coverslips were removed, and tissue sections were washed with 1xPBS and new coverslips were mounted after adding antifade reagent. Confocal microscopy was carried out to detect the VIC fluorescence and images were acquired. Tissue sections incubated only TaqMan master mix lacking cDNA reaction mix was used as negative control (probe only). Fluorescence intensity of MT-ATP8-VIC was analyzed using the ImageJ software.

### Statistical Analysis

Results were expressed as Mean ± SEM. A P value <0.05 was considered to be significant. Statistical analysis was carried out using Unpaired *t*-test or Ordinary One-way ANOVA followed by post hoc using Fisher’s post hoc comparison test for uni-variant intergroup comparison and ordinary Two Way ANOVA with Tukey’s multiple comparisons test was used for bi-variant intergroup comparison. All analyses were carried out using GraphPad Prism Statistical Software, version 7.00 (GraphPad, San Diego, CA, United States). **Sample size:** The sample size in this study was 8 per experimental group (*N* = 8), which was based on our previous study of resuscitative effect of centhaquine on rats after hemorrhagic shock (Lavhale et al., Journal of surgical Research, 179 (2013) 115–124), where a power analysis was conducted using GraphPad Instat-2.00 (GraphPad, San Diego, CA). The power was set to 80% (beta, 0.8), and the level of significance (alpha) used was 0.05. Power analysis indicated that a sample size of at least five per group was required to achieve a power of 80%, when the level of significance alpha is 0.05. **Randomization:** After hemorrhagic shock and I/R rats were randomized to receive either CQ or normal saline. **Blinding:** The histopathology analysis of kidney tissues was performed and interpreted by investigators blinded to the treatment groups of rats.

## Data Availability

The original contributions presented in the study are included in the article/Supplementary Material, further inquiries can be directed to the corresponding authors.

## Abbreviations

CQ, Centhaquine (0.02 mg/kg); AKI, Acute Kidney Injury; MAP, mean arterial Pressure; HR, Heart Rate; KI/R, Kidney Ischemia/Reperfusion; HIF1 1α, Hypoxia Inducible Factor 11α.
