# Supplementary material for: Centhaquine Restores Renal Blood Flow and Protects Tissue Damage After Hemorrhagic Shock and Renal Ischemia
Source: Front Pharmacol. 2021 May 3;12:616253. doi: 10.3389/fphar.2021.616253 (PMC8126696; doi:10.3389/fphar.2021.616253)
Supplement: Supplementary file 1 [file datasheet1.docx]

**Supplementary Materials: -**

**Title - Centhaquine restores renal blood flow and protects tissue damage after hemorrhagic shock and renal ischemia**

**Supplementary Fig. 1. Full western blot images of figure 5 A (HIF1 A and GAPDH).**


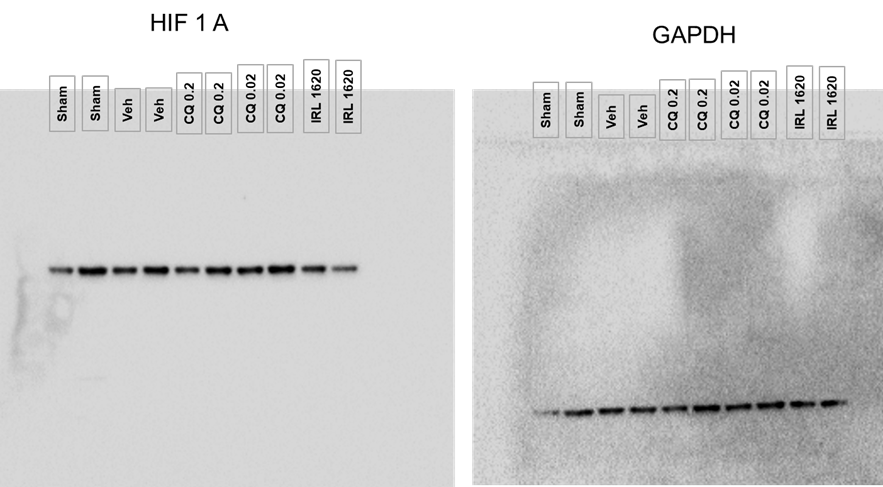


**Supplementary Fig. 2. Full western blot images of figure 5 B (HIF1 B and GAPDH).**


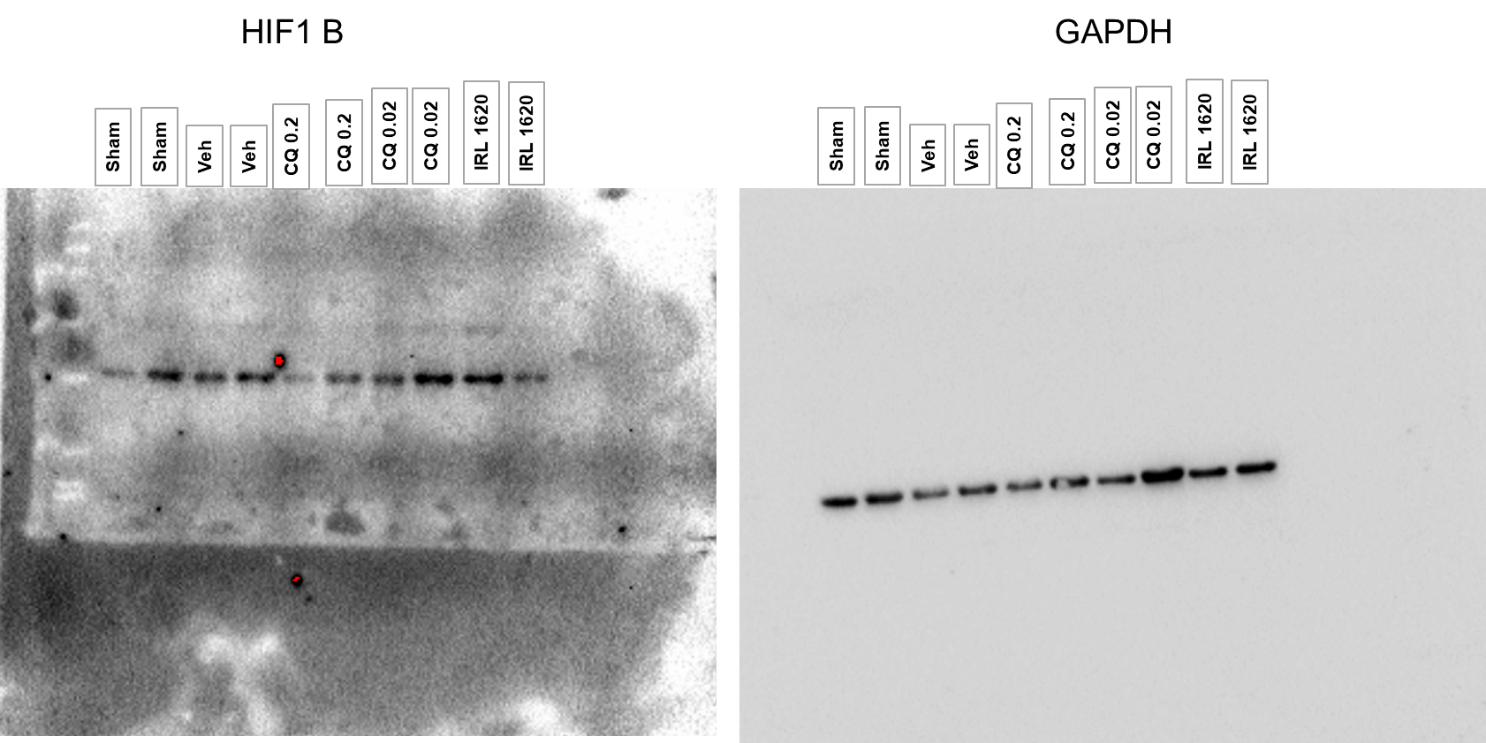


**Supplementary Fig. 3. Full western blot images of figure 6 A (NGAL and GAPDH).**


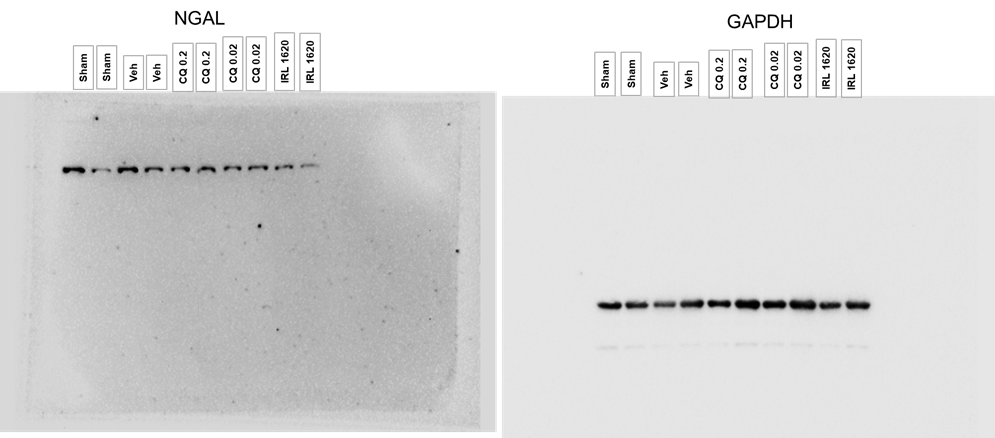


**Supplementary Fig. 4**

**
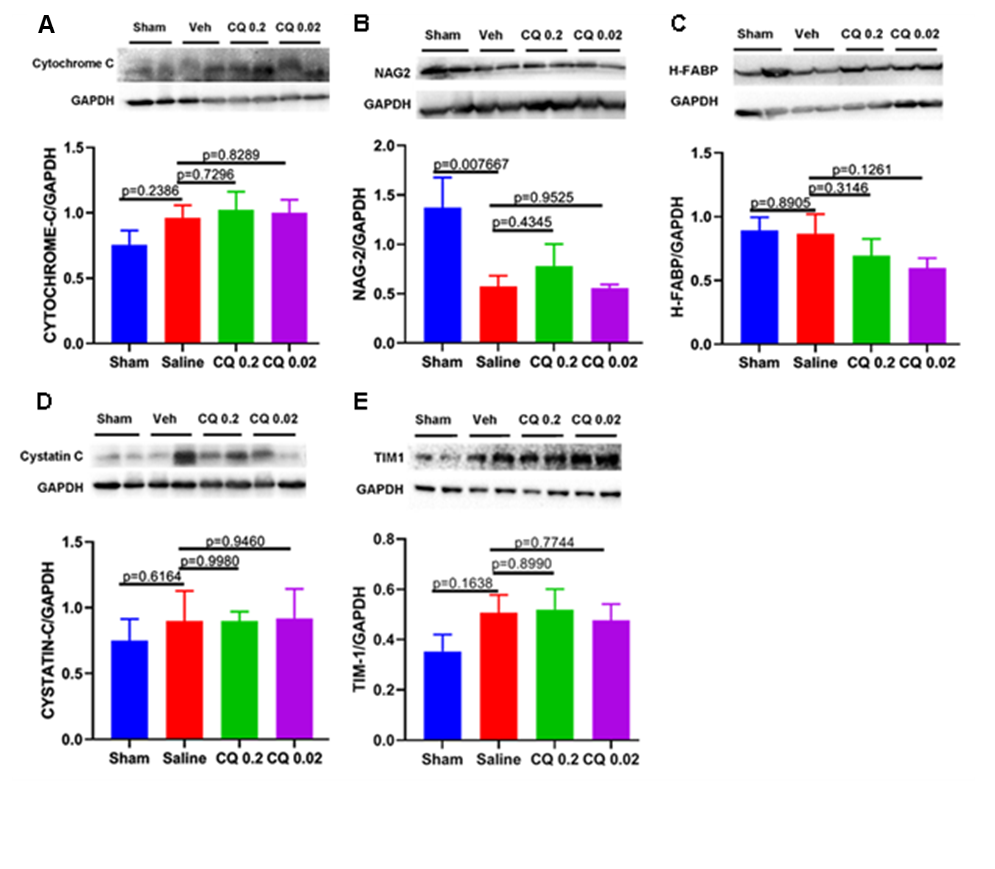
**

**Supplementary Fig. 5.**

**
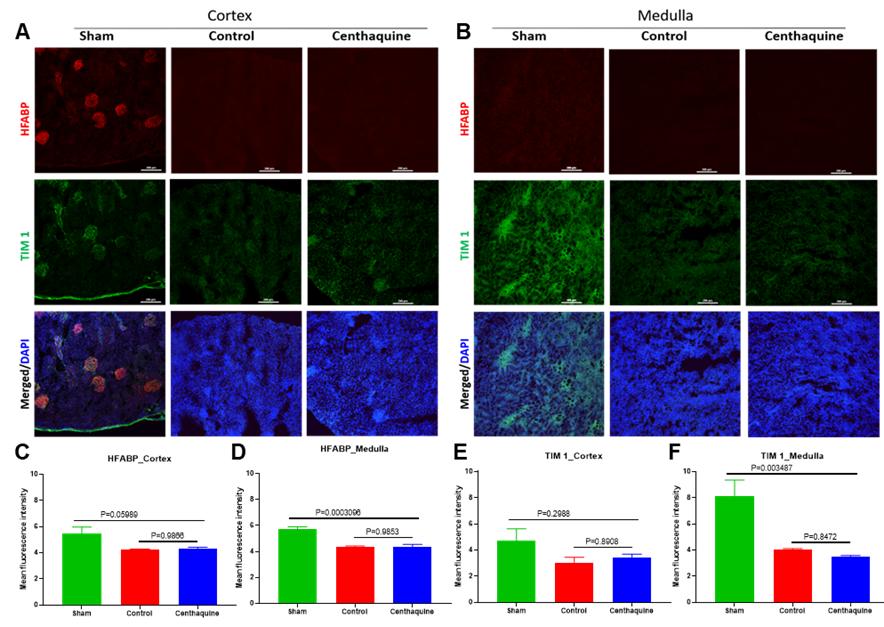
**

**Supplementary Fig. 6.**

**
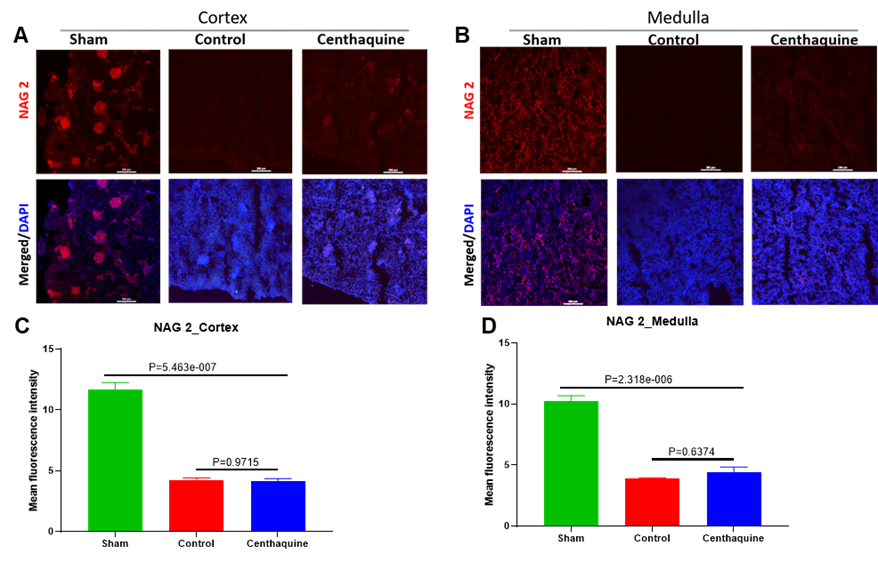
**

**Supplementary Fig. 7.**

**
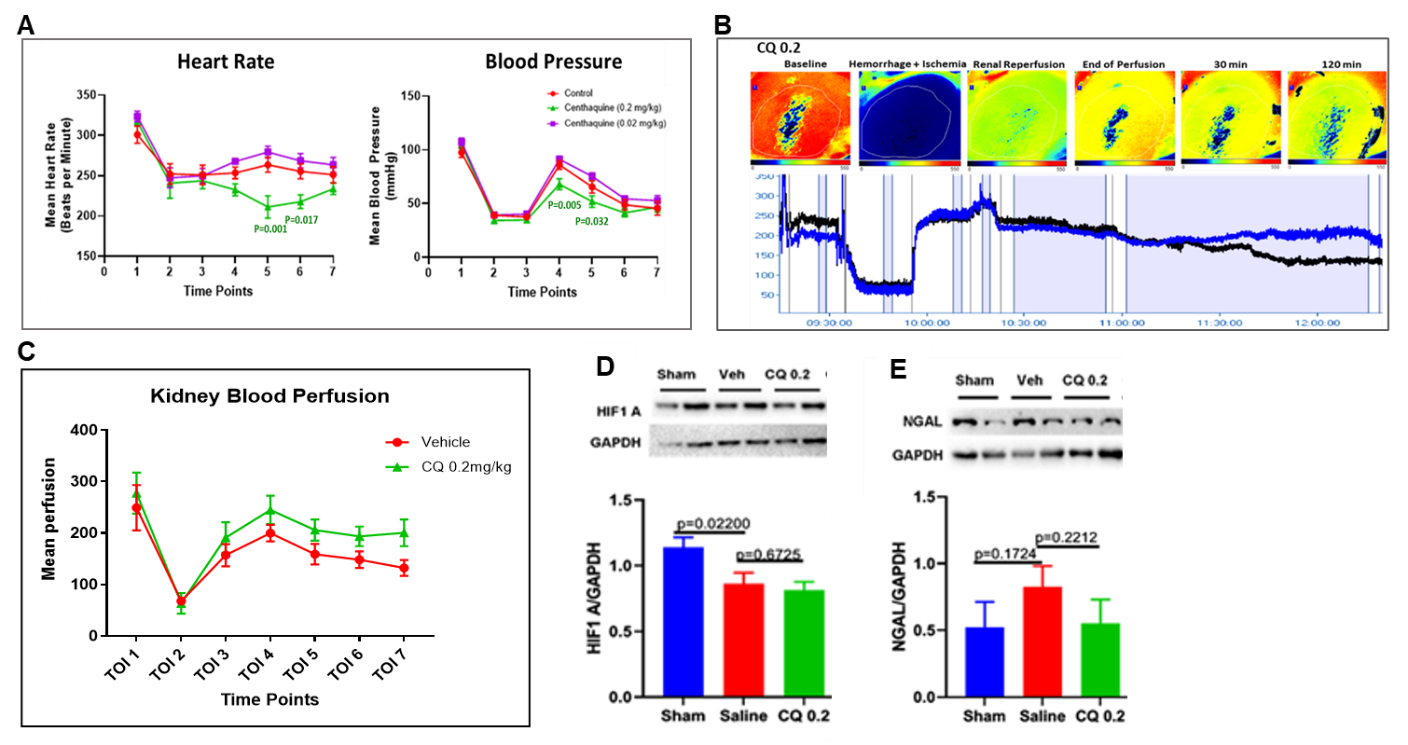
**

**Supplementary Fig. 8.**

**
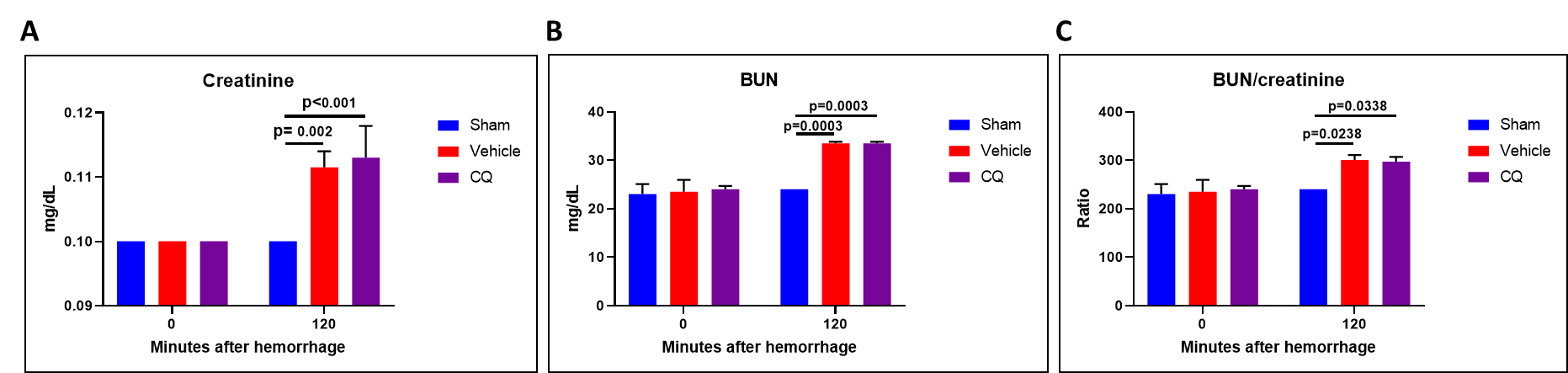
**

**Table 1. Kidney Blood flow data:**

| **Saline** | | | | | | | | | | | | |
| --- | --- | --- | --- | --- | --- | --- | --- | --- | --- | --- | --- | --- |
| **TOI 1** | **Rat S1** | **Rat S2** | **Rat S3** | **Rat S4** | **Rat S5** | **Rat S6** | **Rat S7** |  | **Average** | **SD** | **SEM** |  |
| **TOI 2** | 487.70 | 173.47 | 267.88 | 277.54 | 198.67 | 140.27 | 198.54 |  | **249.15** | **116.01** | **43.85** |  |
| **TOI 3** | 83.97 | 61.05 | 88.29 | 53.89 | 66.42 | 57.12 | 63.61 |  | **67.76** | **13.25** | **5.01** |  |
| **TOI 4** | 76.17 | 154.71 | 138.05 | 136.61 | 207.73 | 134.50 | 251.90 |  | **157.10** | **56.86** | **21.49** |  |
| **TOI 5** | 219.13 | 185.73 | 203.32 | 143.83 | 184.00 | 182.43 | 280.29 |  | **199.82** | **42.32** | **16.00** |  |
| **TOI 6** | 216.84 | 138.31 | 189.38 | 65.55 | 145.40 | 143.94 | 212.66 |  | **158.87** | **52.79** | **19.95** |  |
| **TOI 7** | 215.21 | 134.11 | 137.41 | 91.04 | 131.03 | 133.73 | 195.20 |  | **148.25** | **42.40** | **16.03** |  |
|  |  |  |  |  |  |  |  |  |  |  |  |  |
|  |  |  |  |  |  |  |  |  |  |  |  |  |
| **Centhaquine (0.02mg/kg)** | | | | | | | | | | | | |
| **TOI 1** | **Rat C1** | **Rat C2** | **Rat C3** | **Rat C4** | **Rat C5** | **Rat C6** | **Rat C7** | **Rat C8** | **Average** | **SD** | **SEM** |  |
| **TOI 2** | 300.14 | 255.38 | 267.11 | 256.94 | 179.60 | 294.29 | 191.18 | 273.48 | **252.27** | **44.36** | **15.68** |  |
| **TOI 3** | 82.55 | 51.63 | 60.48 | 55.22 | 61.92 | 81.96 | 66.62 | 70.82 | **66.40** | **11.47** | **4.05** |  |
| **TOI 4** | 180.98 | 171.79 | 213.03 | 201.54 | 143.34 | 194.78 | 189.07 | 168.26 | **182.85** | **21.85** | **7.72** |  |
| **TOI 5** | 266.53 | 230.69 | 296.27 | 234.66 | 270.60 | 249.93 | 248.02 | 259.55 | **257.03** | **21.20** | **7.50** |  |
| **TOI 6** | 251.96 | 226.89 | 285.42 | 213.93 | 226.53 | 216.88 | 215.69 | 236.14 | **234.18** | **24.23** | **8.57** |  |
| **TOI 7** | 235.18 | 206.31 | 231.03 | 211.35 | 247.87 | 206.41 | 196.96 | 159.02 | **211.77** | **27.47** | **9.71** |  |

**Table 2. Heart Rate Data:**

| **HEART RATE (Beats Per Minute)** | | |  |  |  |  |  |
| --- | --- | --- | --- | --- | --- | --- | --- |
|  | **Saline** | | | | | | |
|  | **TOI 1** | **TOI 2** | **TOI 3** | **TOI 4** | **TOI 5** | **TOI 6** | **TOI 7** |
|  | **Heart Rate (BPM)** | **Heart Rate (BPM)** | **Heart Rate (BPM)** | **Heart Rate (BPM)** | **Heart Rate (BPM)** | **Heart Rate (BPM)** | **Heart Rate (BPM)** |
| **Rat S1** | 327.07 | 276.65 | 278.25 | 272.14 | 284.42 | 286.33 | 280.96 |
| **Rat S2** | 254.50 | 216.42 | 209.65 | 223.96 | 227.12 | 227.24 | 218.42 |
| **Rat S3** | 303.62 | 245.25 | 242.51 | 248.87 | 240.21 | 230.15 | 220.24 |
| **Rat S4** | 323.93 | 302.84 | 300.14 | 276.76 | 281.71 | 283.40 | 283.04 |
| **Rat S5** | 283.86 | 207.57 | 218.09 | 244.45 | 272.99 | 248.05 | 255.24 |
| **Rat S7** | 330.99 | 270.53 | 261.30 | 267.67 | 288.90 | 268.96 | 261.25 |
| **Rat S6** | 283.25 | 246.26 | 248.04 | 241.43 | 250.33 | 243.54 | 239.61 |
|  |  |  |  |  |  |  |  |
| **Average** | **301.03** | **252.22** | **251.14** | **253.61** | **263.67** | **255.38** | **251.25** |
| **SD** | **28.53** | **33.80** | **31.98** | **19.19** | **24.30** | **24.32** | **26.41** |
| **SEM** | **11.65** | **13.80** | **13.06** | **7.84** | **9.92** | **9.93** | **10.78** |
|  |  |  |  |  |  |  |  |
|  | **Centhaquine (0.02 mg/kg)** | | | | | | |
|  | **TOI 1** | **TOI 2** | **TOI 3** | **TOI 4** | **TOI 5** | **TOI 6** | **TOI 7** |
|  | **Heart Rate (BPM)** | **Heart Rate (BPM)** | **Heart Rate (BPM)** | **Heart Rate (BPM)** | **Heart Rate (BPM)** | **Heart Rate (BPM)** | **Heart Rate (BPM)** |
| **Rat C1** | 327.07 | 276.65 | 278.25 | 272.14 | 284.42 | 286.33 | 280.96 |
| **Rat C2** | 303.64 | 201.04 | 203.23 | 254.18 | 242.08 | 221.34 | 224.22 |
| **Rat C3** | 352.33 | 206.55 | 228.51 | 281.78 | 296.51 | 282.74 | 271.80 |
| **Rat C4** | 304.90 | 271.57 | 273.16 | 262.18 | 279.02 | 269.75 | 268.95 |
| **Rat C5** | 322.24 | 300.24 | 273.93 | 271.62 | 300.85 | 300.45 | 307.75 |
| **Rat C6** | 347.48 | 239.26 | 249.72 | 276.85 | 290.13 | 267.47 | 260.71 |
| **Rat C7** | 320.46 | 228.13 | 239.35 | 268.41 | 285.81 | 275.78 | 260.11 |
| **Rat C8** | 310.23 | 255.97 | 253.00 | 255.52 | 256.49 | 244.14 | 234.25 |
|  |  |  |  |  |  |  |  |
| **Average** | **323.54** | **247.43** | **249.89** | **267.83** | **279.41** | **268.50** | **263.59** |
| **SD** | **18.31** | **34.97** | **25.85** | **9.85** | **20.19** | **25.11** | **26.13** |
| **SEM** | **5.08** | **9.70** | **7.17** | **2.73** | **5.60** | **6.96** | **7.25** |

Bold values are to demarcate the Average, SD and SEM values

**Table 3. Mean Arterial Pressure Data:**

| **MEAN ARTERIAL PRESSURE (BLOOD PRESSURE, mmHg)** | | | | |  |  |  |
| --- | --- | --- | --- | --- | --- | --- | --- |
|  | **Saline** | | | | | | |
|  | **TOI 1** | **TOI 2** | **TOI 3** | **TOI 4** | **TOI 5** | **TOI 6** | **TOI 7** |
|  | **MAP (mmHg)** | **MAP (mmHg)** | **MAP (mmHg)** | **MAP (mmHg)** | **MAP (mmHg)** | **MAP (mmHg)** | **MAP (mmHg)** |
| **Rat S1** | 117.75 | 45.42 | 37.86 | 100.33 | 76.93 | 52.91 | 58.98 |
| **Rat S2** | 87.81 | 37.79 | 38.72 | 89.25 | 73.52 | 60.90 | 53.51 |
| **Rat S3** | 104.90 | 37.05 | 39.12 | 82.05 | 51.35 | 38.69 | 38.04 |
| **Rat S4** | 87.99 | 33.26 | 35.33 | 75.61 | 46.45 | 34.36 | 19.47 |
| **Rat S5** | 98.27 | 38.11 | 38.75 | 92.72 | 87.05 | 65.30 | 65.40 |
| **Rat S7** | 101.84 | 38.70 | 38.06 | 93.32 | 71.66 | 55.49 | 54.55 |
| **Rat S6** | 84.36 | 42.97 | 35.38 | 69.51 | 52.28 | 34.21 | 29.29 |
|  |  |  |  |  |  |  |  |
| **Average** | **97.56** | **39.04** | **37.60** | **86.11** | **65.61** | **48.84** | **45.61** |
| **SD** | **11.84** | **4.00** | **1.59** | **10.88** | **15.46** | **12.93** | **16.93** |
| **SEM** | **4.83** | **1.63** | **0.65** | **4.44** | **6.31** | **5.28** | **6.91** |
|  |  |  |  |  |  |  |  |
|  | **Centhaquine (0.02 mg/kg)** | | | | | | |
|  | **TOI 1** | **TOI 2** | **TOI 3** | **TOI 4** | **TOI 5** | **TOI 6** | **TOI 7** |
|  | **MAP (mmHg)** | **MAP (mmHg)** | **MAP (mmHg)** | **MAP (mmHg)** | **MAP (mmHg)** | **MAP (mmHg)** | **MAP (mmHg)** |
| **Rat C1** | 117.75 | 45.42 | 37.86 | 100.33 | 76.93 | 52.91 | 58.98 |
| **Rat C2** | 103.65 | 40.58 | 39.42 | 83.77 | 67.45 | 49.67 | 49.06 |
| **Rat C3** | 100.56 | 37.94 | 40.66 | 102.42 | 89.67 | 64.51 | 63.90 |
| **Rat C4** | 92.18 | 38.42 | 42.03 | 78.28 | 62.14 | 48.64 | 57.25 |
| **Rat C5** | 105.38 | 38.97 | 40.13 | 91.71 | 81.95 | 67.65 | 72.15 |
| **Rat C6** | 112.64 | 37.45 | 42.74 | 89.83 | 78.44 | 52.14 | 51.49 |
| **Rat C7** | 105.28 | 38.36 | 39.75 | 88.97 | 71.14 | 56.88 | 42.79 |
| **Rat C8** | 122.58 | 38.84 | 38.91 | 94.86 | 75.49 | 42.99 | 27.30 |
|  |  |  |  |  |  |  |  |
| **Average** | **107.50** | **39.50** | **40.19** | **91.27** | **75.40** | **54.42** | **52.87** |
| **SD** | **9.75** | **2.56** | **1.60** | **8.03** | **8.58** | **8.25** | **13.74** |
| **SEM** | **2.71** | **0.71** | **0.44** | **2.23** | **2.38** | **2.29** | **3.81** |

Bold values are to demarcate the Average, SD and SEM values

**Table 4. Blood Gas Components Values of Vehicle Rats:**

| **Blood Gas Components of Vehicle Rats** | | | | | | | | | | |
| --- | --- | --- | --- | --- | --- | --- | --- | --- | --- | --- |
| **Baseline** | |  |  |  |  |  |  |  |  |  |
|  | **Rat S1** | **Rat S2** | **Rat S3** | **Rat S4** | **Rat S5** | **Rat S6** | **Rat S7** | **Average** | **SD** | **SEM** |
| **pH** | 7.24 | 7.22 | 7.25 | 7.27 | 7.29 | 7.24 | 7.26 | 7.25 | 0.02 | 0.01 |
| **pCO2 (mmHg)** | 56 | 54 | 49 | 48 | 49 | 51 | 57 | 52.00 | 3.65 | 1.38 |
| **P02 (mmHg)** | 112 | 122 | 132 | 122 | 119 |  |  | 121.40 | 7.20 | 2.72 |
| **Na+ (mmol/L)** | 142 | 136 | 137 | 135 | 134 | 128 |  | 135.33 | 4.55 | 1.72 |
| **K+ (mmol/L** | 3.9 | 3.9 | 3.8 | 3.6 | 4 | 4.5 | 3.9 | 3.94 | 0.28 | 0.10 |
| **Ca++ (mmol/L)** | 1.21 | 1.25 | 1.11 | 1.14 | 1.23 | 1.19 | 1.17 | 1.19 | 0.05 | 0.02 |
| **Lac (mmol/L)** | 3.6 | 3.7 | 1.6 | 3 | 2.8 | 3.4 | 2.9 | 3.00 | 0.71 | 0.27 |
| **Hct (%)** | 48 | 50 | 46 | 51 | 56 | 60 | 55 | 52.29 | 4.92 | 1.86 |
| **HCO3std (mmol/L)** | 21.6 | 20 | 20.3 | 20.9 | 22.1 |  |  | 20.98 | 0.88 | 0.33 |
| **TCO2 (mmol/L)** | 25.7 | 23.8 | 23 | 23.5 | 25.1 | 23.5 | 27.3 | 24.56 | 1.55 | 0.58 |
| **THbc (g/dL)** | 14.9 | 15.5 | 14.3 | 15.8 | 17.4 | 18.6 | 17.1 | 16.23 | 1.53 | 0.58 |
|  |  |  |  |  |  |  |  |  |  |  |
| **After Hemorrhage** | |  |  |  |  |  |  |  |  |  |
|  | **Rat S1** | **Rat S2** | **Rat S3** | **Rat S4** | **Rat S5** | **Rat S6** | **Rat S7** | **Average** | **SD** | **SEM** |
| **pH** |  |  | 7.21 | 7.2 | 7.05 | 7.18 | 7.21 | 7.17 | 0.07 | 0.03 |
| **pCO2 (mmHg)** |  |  | 47 | 45 | 51 | 47 | 49 | 47.80 | 2.28 | 1.02 |
| **P02 (mmHg)** |  |  | 188 | 137 | 140 |  |  | 155.00 | 28.62 | 12.80 |
| **Na+ (mmol/L)** |  |  | 130 | 131 | 131 | 122 |  | 127.70 | 3.30 | 0.03 |
| **K+ (mmol/L** |  |  | 4.9 | 5.1 | 5.3 | 5.3 | 4.7 | 5.06 | 0.26 | 0.12 |
| **Ca++ (mmol/L)** |  |  | 1.19 | 1.26 | 1.33 | 1.19 | 1.17 | 1.23 | 0.07 | 0.03 |
| **Lac (mmol/L)** |  |  | 4.6 | 6.9 | 9.2 | 4.8 | 6 | 6.30 | 1.87 | 0.84 |
| **Hct (%)** |  |  | 43 | 49 | 51 | 51 | 44 | 47.60 | 3.85 | 1.72 |
| **HCO3std (mmol/L)** |  |  | 18 | 16.9 | 12 |  |  | 15.63 | 3.19 | 1.43 |
| **TCO2 (mmol/L)** |  |  | 20.2 | 19 | 15.7 | 18.9 | 21.1 | 18.98 | 2.05 | 0.92 |
| **THbc (g/dL)** |  |  | 13.3 | 15.2 | 15.8 | 15.8 | 13.6 | 14.74 | 1.21 | 0.54 |
|  |  |  |  |  |  |  |  |  |  |  |
| 30 min After Resuscitation | |  |  |  |  |  |  |  |  |  |
|  | **Rat S1** | **Rat S2** | **Rat S3** | **Rat S4** | **Rat S5** | **Rat S6** | **Rat S7** | **Average** | **SD** | **SEM** |
| **pH** | 7.28 | 7.2 | 7.17 | 7.17 | 7.35 | 7.15 | 7.17 | 7.21 | 0.07 | 0.03 |
| **pCO2 (mmHg)** | 34 | 42 | 53 | 49 | 25 | 58 | 64 | 46.43 | 13.70 | 5.18 |
| **P02 (mmHg)** | 166 | 141 | 152 | 110 | 137 |  |  | 141.20 | 20.75 | 7.84 |
| **Na+ (mmol/L)** | 134 | 133 | 131 | 133 | 130 | 128 |  | 131.42 | 2.02 | 0.03 |
| **K+ (mmol/L** | 4.8 | 4.3 | 4.6 | 4.9 | 4.7 | 4.5 | 4 | 4.54 | 0.31 | 0.12 |
| **Ca++ (mmol/L)** | 1.15 | 1.24 | 1.18 | 1.26 | 0.97 | 1.18 | 1.12 | 1.16 | 0.10 | 0.04 |
| **Lac (mmol/L)** | 5.6 | 7.1 | 2.7 | 4.7 | 2.6 | 2.4 | 2.4 | 3.93 | 1.89 | 0.71 |
| **Hct (%)** | 50 | 41 | 41 | 47 | 58 | 51 | 49 | 48.14 | 5.96 | 2.25 |
| **HCO3std (mmol/L)** | 17.4 | 16.2 | 17.6 | 16.6 | 17.6 |  |  | 17.08 | 0.64 | 0.24 |
| **TCO2 (mmol/L)** | 17 | 17.7 | 20.9 | 19.4 | 14.6 | 22 | 25.3 | 19.56 | 3.54 | 1.34 |
| **THbc (g/dL)** | 15.5 | 12.7 | 12.7 | 14.6 | 18 | 15.8 | 15.2 | 14.93 | 1.85 | 0.70 |
|  |  |  |  |  |  |  |  |  |  |  |
| 120 min After Resuscitation | |  |  |  |  |  |  |  |  |  |
|  | **Rat S1** | **Rat S2** | **Rat S3** | **Rat S4** | **Rat S5** | **Rat S6** | **Rat S7** | **Average** | **SD** | **SEM** |
| **pH** |  | 7.15 | 7.21 | 7.17 | 7.25 | 7.16 | 7.18 | 7.19 | 0.04 | 0.02 |
| **pCO2 (mmHg)** |  | 54 | 41 | 28 | 27 | 43 | 55 | 41.33 | 12.11 | 4.94 |
| **P02 (mmHg)** |  | 135 | 188 | 149 | 159 |  |  | 157.75 | 22.44 | 9.16 |
| **Na+ (mmol/L)** |  | 131 | 130 | 137 | 133 | 123 |  | 129.36 | 3.64 | 0.02 |
| **K+ (mmol/L** |  | 5.3 | 5.8 | 4.9 | 4.9 | 5.6 | 5.1 | 5.27 | 0.37 | 0.15 |
| **Ca++ (mmol/L)** |  | 1.25 | 1.18 | 1.11 | 1.23 | 1.18 | 1.14 | 1.18 | 0.05 | 0.02 |
| **Lac (mmol/L)** |  | 3.4 | 3.9 | 6.7 | 4.7 | 3.7 | 2.3 | 4.12 | 1.49 | 0.61 |
| **Hct (%)** |  | 48 | 40 | 39 | 53 | 54 | 50 | 47.33 | 6.44 | 2.63 |
| **HCO3std (mmol/L)** |  | 16.8 | 16.5 | 11.8 | 14.4 |  |  | 14.88 | 2.31 | 0.94 |
| **TCO2 (mmol/L)** |  | 20.5 | 17.7 | 11.1 | 12.6 | 16.6 | 22.2 | 16.78 | 4.33 | 1.77 |
| **THbc (g/dL)** |  | 14.9 | 12.4 | 12.1 | 16.4 | 16.7 | 15.5 | 14.67 | 1.98 | 0.81 |

**Table 5. Blood Gas Components Values of CQ Rats:**

| **Blood Gas Components of CQ Rats (Centhaquin 0.02 mg/kg)** | | | | | | | | | | | | |
| --- | --- | --- | --- | --- | --- | --- | --- | --- | --- | --- | --- | --- |
| **Baseline** | |  |  |  |  |  |  |  |  |  |  |  |
|  | **Rat C1** | **Rat C2** | **Rat C3** | **Rat C4** | **Rat C5** | **Rat C6** | **Rat C7** | **Rat C8** | **Average** | **SD** | **SEM** |  |
| **pH** | 7.29 | 7.27 | 7.32 | 7.25 | 7.28 | 7.3 | 7.26 | 7.23 | 7.28 | 0.03 | 0.01 |  |
| **pCO2 (mmHg)** | 49 | 51 | 47 | 52 | 51 | 53 | 49 | 53 | 50.63 | 2.13 | 0.75 |  |
| **P02 (mmHg)** |  |  |  |  |  | 110 | 116 | 121 | 115.67 | 5.51 | 1.95 |  |
| **Na+ (mmol/L)** | 135 | 134 | 134 | 132 | 133 | 136 | 137 | 135 | 134.50 | 1.60 | 0.57 |  |
| **K+ (mmol/L** | 3.6 | 3.6 | 4.2 | 3.7 | 3.8 | 3.8 | 3.3 | 4 | 3.75 | 0.27 | 0.10 |  |
| **Ca++ (mmol/L)** | 1.18 | 1.2 | 1.19 | 1.2 | 1.21 | 1.16 | 1.09 | 1.16 | 1.17 | 0.04 | 0.01 |  |
| **Lac (mmol/L)** | 3.1 | 2.8 | 2.8 | 4.1 | 3.5 | 3 | 3.1 | 2.9 | 3.16 | 0.44 | 0.16 |  |
| **Hct (%)** | 58 | 53 | 60 | 53 | 57 | 58 | 48 | 59 | 55.75 | 4.06 | 1.44 |  |
| **HCO3std (mmol/L)** |  |  |  |  |  | 23.8 | 20.7 | 20.1 | 21.53 | 1.99 | 0.70 |  |
| **TCO2 (mmol/L)** | 25.1 | 25 | 25.6 | 24.4 | 25.6 | 27.7 | 23.5 | 23.8 | 25.09 | 1.31 | 0.46 |  |
| **THbc (g/dL)** | 18 | 16.4 | 18.6 | 16.4 | 17.7 | 18 | 14.9 | 18.3 | 17.29 | 1.27 | 0.45 |  |
|  |  |  |  |  |  |  |  |  |  |  |  |  |
| **After Hemorrhage** | |  |  |  |  |  |  |  |  |  |  |  |
|  | **Rat C1** | **Rat C2** | **Rat C3** | **Rat C4** | **Rat C5** | **Rat C6** | **Rat C7** | **Rat C8** | **Average** | **SD** | **SEM** |  |
| **pH** | 7.26 | 7.21 | 7.26 | 7.21 | 7.24 | 7.22 | 7.21 | 7.12 | 7.22 | 0.04 | 0.02 |  |
| **pCO2 (mmHg)** | 31 | 44 | 35 | 46 | 38 | 48 | 53 | 46 | 42.63 | 7.33 | 2.59 |  |
| **P02 (mmHg)** |  |  |  |  |  | 125 | 128 | 145 | 132.67 | 10.79 | 3.81 |  |
| **Na+ (mmol/L)** | 132 | 128 | 127 | 128 | 125 | 132 | 127 | 128 | 128.38 | 2.45 | 0.86 |  |
| **K+ (mmol/L** | 4.7 | 4.5 | 5 | 4.3 | 4.6 | 4.5 | 4.3 | 5 | 4.61 | 0.27 | 0.10 |  |
| **Ca++ (mmol/L)** | 1.16 | 1.28 | 1.16 | 1.24 | 1.22 | 1.22 | 1.25 | 1.26 | 1.22 | 0.04 | 0.02 |  |
| **Lac (mmol/L)** | 8.1 | 7.2 | 7.4 | 7.2 | 8.5 | 7.3 | 5.5 | 8.3 | 7.44 | 0.94 | 0.33 |  |
| **Hct (%)** | 45 | 44 | 49 | 41 | 42 | 48 | 43 | 52 | 45.50 | 3.82 | 1.35 |  |
| **HCO3std (mmol/L)** |  |  |  |  |  | 18.5 | 19.4 | 13.8 | 17.23 | 3.01 | 1.06 |  |
| **TCO2 (mmol/L)** | 14.9 | 19 | 16.8 | 19.8 | 17.5 | 21.1 | 22.8 | 16.4 | 18.54 | 2.63 | 0.93 |  |
| **THbc (g/dL)** | 14 | 13.6 | 15.2 | 12.7 | 13 | 14.9 | 13.3 | 16.1 | 14.10 | 1.19 | 0.42 |  |
|  |  |  |  |  |  |  |  |  |  |  |  |  |
| **30 min After Resuscitation** | |  |  |  |  |  |  |  |  |  |  |  |
|  | **Rat C1** | **Rat C2** | **Rat C3** | **Rat C4** | **Rat C5** | **Rat C6** | **Rat C7** | **Rat C8** | **Average** | **SD** | **SEM** |  |
| **pH** | 7.21 | 7.2 | 7.19 | 7.22 | 7.24 | 7.24 | 7.22 | 7.16 | 7.21 | 0.03 | 0.01 |  |
| **pCO2 (mmHg)** | 49 | 56 | 52 | 50 | 50 | 56 | 60 | 53 | 53.25 | 3.81 | 1.35 |  |
| **P02 (mmHg)** |  |  |  |  |  | 114 | 116 | 131 | 120.33 | 9.29 | 3.29 |  |
| **Na+ (mmol/L)** | 133 | 128 | 126 |  | 127 | 131 | 128 | 136 | 129.86 | 3.63 | 1.28 |  |
| **K+ (mmol/L** | 3.8 | 4.3 | 4.9 | 4.1 | 4.6 | 4.2 | 4.3 | 3.9 | 4.26 | 0.36 | 0.13 |  |
| **Ca++ (mmol/L)** | 1.16 | 1.18 | 1.23 |  | 1.22 | 1.16 | 1.25 | 1.12 | 1.19 | 0.05 | 0.02 |  |
| **Lac (mmol/L)** | 2.6 | 2.8 | 2.2 | 3.8 | 4.4 | 2.5 | 2.7 | 2.6 | 2.95 | 0.75 | 0.26 |  |
| **Hct (%)** | 52 | 46 | 55 |  | 49 | 51 | 49 | 51 | 50.43 | 2.82 | 1.00 |  |
| **HCO3std (mmol/L)** |  |  |  |  |  | 21.5 | 21.6 | 16.9 | 20.00 | 2.69 | 0.95 |  |
| **TCO2 (mmol/L)** | 21.1 | 23.6 | 21.5 | 22 | 22.9 | 25.7 | 26.4 | 20.5 | 22.96 | 2.15 | 0.76 |  |
| **THbc (g/dL)** | 16.1 | 14.3 | 17.1 |  | 15.2 | 15.8 | 15.2 | 15.8 | 15.64 | 0.87 | 0.31 |  |
|  |  |  |  |  |  |  |  |  |  |  |  |  |
| **120 min After Resuscitation** | |  |  |  |  |  |  |  |  |  |  |  |
|  | **Rat C1** | **Rat C2** | **Rat C3** | **Rat C4** | **Rat C5** | **Rat C6** | **Rat C7** | **Rat C8** | **Average** | **SD** | **SEM** |  |
| **pH** | 7.2 | 7.18 |  | 7.2 | 7.23 | 7.2 | 7.17 | 7.11 | 7.18 | 0.04 | 0.01 |  |
| **pCO2 (mmHg)** | 48 | 55 |  | 52 | 49 | 55 | 61 | 49 | 52.71 | 4.64 | 1.64 |  |
| **P02 (mmHg)** |  |  |  |  |  | 95 | 125 | 132 | 117.33 | 19.66 | 6.95 |  |
| **Na+ (mmol/L)** | 128 | 131 |  | 127 | 127 | 127 | 125 | 129 | 127.71 | 1.89 | 0.67 |  |
| **K+ (mmol/L** | 4.7 | 4.2 |  | 4.4 | 4.6 | 4.5 | 4.8 | 5.8 | 4.71 | 0.52 | 0.18 |  |
| **Ca++ (mmol/L)** | 1.13 | 1.17 |  | 1.21 | 1.23 | 1.15 | 1.21 | 1.22 | 1.19 | 0.04 | 0.01 |  |
| **Lac (mmol/L)** | 2.1 | 2.5 |  | 2.2 | 1.6 | 2.4 | 2.1 | 5.1 | 2.57 | 1.15 | 0.41 |  |
| **Hct (%)** | 53 | 47 |  | 43 | 51 | 50 | 47 | 53 | 49.14 | 3.67 | 1.30 |  |
| **HCO3std (mmol/L)** |  |  |  |  |  | 19.2 | 19.3 | 14 | 17.50 | 3.03 | 1.07 |  |
| **TCO2 (mmol/L)** | 20.3 | 22.2 |  | 21.9 | 22 | 23.2 | 24.2 | 17.1 | 21.56 | 2.30 | 0.81 |  |
| **THbc (g/dL)** | 16.4 | 14.6 |  | 13.3 | 15.8 | 15.5 | 14.6 | 16.4 | 15.23 | 1.13 | 0.40 |  |

**Figure legends (Supplementary Materials)**

**Supplementary Fig. 1. Full western blot images of HIF 1 A and GAPDH (loading control, developed on the same blot of HIF1 A after stripping off the blot).** The bands in blue rectangular area were cropped and assembled to prepare the representative western blot images of HIF-1Α and GAPDH in the Fig. 5A.

**Supplementary Fig. 2. Full western blot images of HIF 1 B and GAPDH (loading control, developed on the same blot of HIF1 A after stripping off the blot).** The bands in blue rectangular area were cropped and assembled to prepare the representative western blot images of HIF 1B and GAPDH in the Fig. 5B.

**Supplementary Fig. 3. Full western blot images of NGAL and GAPDH (loading control, developed on the same blot of NGAL after stripping off the blot).** The bands in blue rectangular area were cropped and assembled to prepare the representative western blot images of NGAL and GAPDH in the Fig. 6.

**Supplementary Fig. 4. Representative western blot images of cytochrome C and other kidney damage markers (NAG 2, HFABP, Cystatin C and TIM 1), and their normalized densitometry graphs A-E.** The error bar represents mean ± SEM. N=4.

**Supplementary Fig. 5. Representative images of immunofluorescence of HFABP (red) and TIM 1 (green) in cortex (A) and medulla (B) in sham, vehicle and CQ**. C-D, graphs of mean fluorescence intensity of HFAPB in cortex (C) and medulla (D), and of TIM 1 in cortex (E) and medulla (F). N=4. Nuclei in all immunofluorescence microscopy images were stained with DAPI (blue).

**Supplementary Fig. 6. Representative images of immunofluorescence of NAG 2 (red) in cortex (A) and medulla (B), in sham, vehicle and CQ**. C-D, graphs of mean fluorescence intensity of NAG 2 in cortex (C) and medulla (D). N=4. Nuclei in all immunofluorescence microscopy images were stained with DAPI (blue).

**Supplementary Fig. 7. Effect of high dose CQ (0.2 mg/kg) on cardiovascular actions, blood perfusion and hypoxia response. A,** graphs showing change cardiovascular actions (MAP and HR) in vehicle (red), low dose CQ (purple) and high dose CQ (green) at different time points (1 – baseline, 2 - after hemorrhage, 3 - after de-clamping renal arteries, 4 - 0 min post-perfusion, 5 - 30 min post perfusion, 6 - 90 min post-perfusion and 7 - 120 min post-perfusion). Data represents mean ± SEM values. N= 7 for vehicle and N= 8 for CQ. Statistical analysis – unpaired T-tests. **B,** representative images of blood flow in kidneys of CQ 0.2 (high dose). Upper panel shows kidney images obtained from PeriCam PSI blood flow imaging system. Red represents highest blood flow and blue represents the lowest. The lower panel show the blood flow graphs – Blue line indicates blood flow in complete imaged area, Black line indicates blood flow in the kidney. N= 7. **C**, a graphical representation of kidney blood flow at different time points (1 – baseline, 2 - after hemorrhage, 3 - after de-clamping renal arteries, 4 - 0 min post-perfusion, 5 - 30 min post-perfusion, 6 - 90 min post-perfusion and 7 - 120 min post-perfusion). Data represents mean ± SEM values. Statistical analysis – unpaired T-tests. **D** and **E,** upper panels show western blots of HIF-1α and NGAL in kidney tissues of sham, vehicle (saline) and CQ 0.2. GAPDH – loading control. Lower panels show their respective densitometry graphs. N=4. The error bar represents mean ± SEM. Statistical analysis- Ordinary One Way ANOVA and Fisher’s test.

**Supplementary Fig. 8. Effect of CQ on the levels of creatinine and BUN in plasma. A-C**, graphs of estimated creatinine (A), BUN (B) and BUN / creatinine ratio (C) in plasma of rats at baseline (before hemorrhage) and at 120 min of resuscitation. N=4. Statistical analysis – Ordinary Two Way ANOVA with Tukey’s multiple comparisons test.
